# NUSAP1 Recruits DAXX to Suppress HIF‐Driven Triple‐Negative Breast Cancer Progression

**DOI:** 10.1002/advs.202513380

**Published:** 2025-11-03

**Authors:** Yating Du, Jingjing Wang, Min Wang, Yao Zhang, Miaomiao Zheng, Huiyan Li, Xuemeng Wang, Huanran Sun, Kexin Tang, Changliang Shan, Qiang Zhao, Xiaoqian Meng, Yijie Wang, Jun Zhou, Yan Chen

**Affiliations:** ^1^ Collaborative Innovation Center of Cell Biology in Universities of Shandong Center for Cell Structure and Function College of Life Sciences Modern Industry Institute of Biomedicine Shandong Normal University Jinan 250014 China; ^2^ School of Medicine Jinan University Guangzhou 510630 China; ^3^ College of Pharmacy Nankai University Tianjin 300350 China; ^4^ School of Mathematics and Statistics Shandong Normal University Jinan 250014 China; ^5^ College of Life Sciences Nankai University Tianjin 300071 China

**Keywords:** DAXX, HIF, hypoxia, NUSAP1, triple‐negative breast cancer

## Abstract

Nucleolar and spindle‐associated protein 1 (NUSAP1) is critical for cancer progression. However, its function in cancers is context‐dependent, and emerging evidence indicates that NUSAP1 possesses tumor suppressor functions, although the underlying mechanisms remain uncharted. In this study, NUSAP1 depletion is found to significantly increases the proliferation, migration, and invasion of triple‐negative breast cancer (TNBC) cells in vitro and promotes TNBC progression in vivo, suggesting that NUSAP1 is a tumor suppressor in TNBC. Mechanistically, NUSAP1 bridges HIFα and the transcriptional repressor death domain‐associated protein (DAXX) through its microtubule‐associated domain (MAD) to recruit the methyltransferase SETDB1, thereby attenuating HIF transcriptional activity and the expression of its target genes by depositing the H3K9me3 repressive mark on hypoxia response elements (HREs). Intriguingly, an engineered MAD of NUSAP1, designated as Tumor Suppressor‐MAD (TS‐MAD), is developed, which effectively abrogates HIF transcriptional activity by bridging the DAXX‐HIF interaction, consequently inhibiting HIF‐driven TNBC progression. Moreover, *NUSAP1* is identified as a novel HIF‐repressed gene in TNBC cells, and its expression level shows a negative correlation with clinical outcomes in TNBC patients. These findings establish an HIF‐NUSAP1 double‐negative feedback loop in TNBC and validate TS‐MAD as a potential therapeutic strategy for HIF‐driven cancer.

## Graphical abstract

A double‐negative feedback loop is identified in TNBC where NUSAP1 bridges HIF and DAXX via its microtubule‐associated domain (MAD) to recruit SETDB1, repressing HIF transcriptional activity by depositing the H3K9me3 repressive mark on HIF target genes, while HIF itself suppresses NUSAP1. Therapeutically, the TS‐MAD (Tumor Suppressor‐MAD) miniprotein mimics NUSAP1's function, offering a promising strategy to intervene in HIF‐driven TNBC.

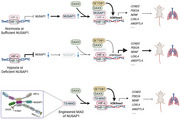

## 1 Introduction

Nucleolar Spindle‐Associated Protein 1 (NUSAP1) was initially characterized as a microtubule‐associated protein (MAP) essential for mitotic spindle assembly and chromosomal segregation.^[^ ^1^, ^2^ ^]^ Intriguingly, during interphase, NUSAP1 predominantly localizes to the nucleus. Further investigations have demonstrated that NUSAP1 possesses the capacity to associate with and accumulate on chromatin,^[^ ^1^, ^2^ ^]^ indicating a potential role for NUSAP1 in transcriptional regulation. Emerging evidence supports this notion. In lung cancer, tumor cell‐secreted lactate induces NUSAP1 nuclear translocation in cancer‐associated fibroblasts (CAFs), where it recruits the JUNB‐FRA1‐FRA2 transcriptional complex to activate *Desmin* (*DES*) transcription, thereby promoting tumor growth.^[^ ^3^ ^]^ In pancreatic ductal adenocarcinoma (PDAC), NUSAP1 forms a complex with c‐MYC and HIF1α to transactivate *Lactate dehydrogenase A* (*LDHA*), driving glycolysis and metastasis.^[^ ^4^ ^]^ Beyond transcriptional regulation, NUSAP1 also promotes the proliferation and metastasis of cervical cancer, astrocytoma, breast cancer, and glioblastoma cells by activating Wnt/β‐catenin,^[^ ^5^ ^]^ Hedgehog,^[^ ^6^ ^]^ AMP‐activated protein kinase (AMPK),^[^ ^7^ ^]^ and ataxia telangiectasia and Rad3‐related (ATR)^[^ ^8^ ^]^ signaling pathways, respectively. These findings suggest that NUSAP1 possesses oncogenic potential. Paradoxically, other studies have reported a tumor‐suppressive role of NUSAP1. NUSAP1 expression is downregulated in cervical cancer cells, leading to poor clinical outcomes.^[^ ^9^ ^]^ NUSAP1 is degraded by the ubiquitin ligase anaphase‐promoting complex (APC/C),^[^ ^10^, ^11^ ^]^ thereby relieving NUSAP1‐mediated cell cycle arrest in MCF‐7 breast cancer and HCT116 colon cancer cells.^[^ ^11^ ^]^ Notably, nuclear localization is required for NUSAP1 to trigger cell cycle arrest.^[^ ^11^ ^]^ Consistently, nuclear‐localized NUSAP1 triggers apoptosis in zebrafish.^[^ ^12^ ^]^ NUSAP1 overexpression inhibits HepG2 cell proliferation, migration, and invasion.^[^ ^13^ ^]^ These conflicting observations suggest that NUSAP1's function depends on its cellular context and subcellular localization. Although nuclear NUSAP1 appears critical for its functions, the precise molecular mechanisms remain unresolved.

Hypoxia, a common phenomenon in solid tumors, is associated with tumor aggressiveness, metastasis, and drug resistance and is therefore considered a hallmark of solid tumors.^[^ ^14^, ^15^ ^]^ Hypoxia‐inducible factors (HIFs), comprising an oxygen‐sensitive HIFα subunit (HIF1α or HIF2α) and a constitutively expressed HIFβ subunit (HIF1β), are the master regulators of cellular adaptation to hypoxia.^[^ ^15^, ^16^ ^]^ Under aerobic conditions, HIFα undergoes proteasomal degradation mediated by von Hippel‐Lindau (VHL) E3 ubiquitin ligase.^[^ ^15^ ^]^ In the hypoxic tumor microenvironment, HIFα is stabilized and translocated into the nucleus, heterodimerizes with HIF1β,^[^ ^15^ ^]^ and binds to hypoxia‐response elements (HREs) of HIF target genes to stimulate transcription of numerous oncogenes, whose protein products control multiple cancer processes, including metastasis, metabolic rewiring, immune evasion, and pluripotency. It is worth noting that HIFs can also mediate the transcriptional repression of specific genes in cancer cells, such as *DAXX* (*Death domain‐associated protein*) in lung cancer cells,^[^ ^17^ ^]^ *CDH1* (*Cadherin‐1*) in renal cell carcinoma (RCC) cells and pancreatic carcinoma cells,^[^ ^18^, ^19^ ^]^ *AFP* (*α‐fetoprotein*) in hepatoma cells,^[^ ^20^ ^]^ and *CAD* (*Carbamoyl phosphate synthetase 2‐aspartate transcarbamylase‐dihydroorotase*) in neuroblastoma and colon carcinoma cells.^[^ ^21^ ^]^ However, the repertoire of HIF‐repressed genes remains limited, and their functional significance and regulatory mechanisms in hypoxic cancer microenvironments remain poorly understood. HIFα protein levels are significantly increased in almost all solid tumors and subsequently lead to poor clinical outcomes.^[^ ^14^ ^]^ Thus, HIFα is considered a biomarker and therapeutic target for cancer diagnosis and treatment. However, targeting HIFs in cancer therapy remains challenging, and currently available HIF inhibitors have limited clinical utility owing to systemic side effects.^[^ ^22^ ^]^

In this study, we demonstrated that NUSAP1 scaffolds the interaction between HIF and the transcriptional corepressor DAXX through its microtubule‐associated domain (MAD) and recruits the methyltransferase SETDB1 in the nucleus to attenuate HIF transcriptional activity by depositing H3K9me3 on hypoxia response elements (HREs), thereby inhibiting the transcription of HIF target genes that drive metastasis and proliferation. This NUSAP1‐mediated suppression of HIF signaling significantly inhibits TNBC growth and metastasis. Importantly, the engineered Tumor Suppressor‐MAD (TS‐MAD) mini‐protein recapitulates this tumor‐suppressive mechanism by disrupting HIF transcriptional activity and potently inhibiting TNBC progression. Additionally, we identified *NUSAP1* as a novel HIF‐repressing gene in TNBC, and low levels of NUSAP1 expression correlate with poor clinical outcomes in TNBC patients. Our findings establish a double‐negative feedback loop between NUSAP1 and HIF that regulates TNBC progression. These data not only reveal a novel regulatory layer of HIF signaling but also highlight the therapeutic potential of TS‐MAD in HIF‐driven cancers.

## 2 Results

### 2.1 Nusap1 Functions as a Tumor Suppressor in Triple‐Negative Breast Cancer (Tnbc) Cells

**Figure 1:**
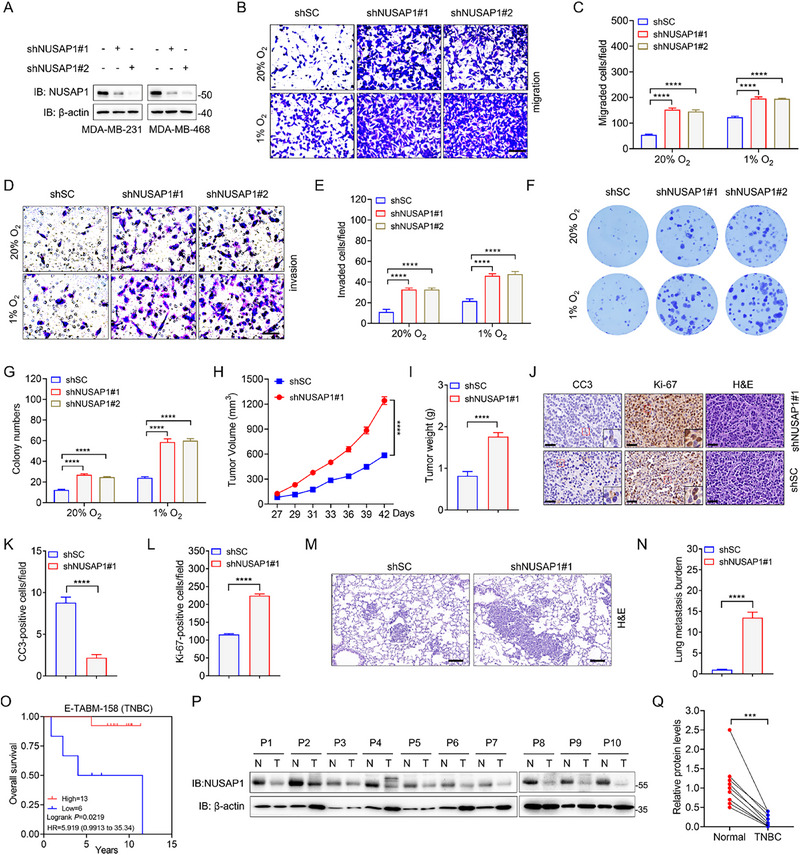
NUSAP1 exhibits tumor‐suppressive properties in TNBC. A) Knockdown efficiency of shNUSAP1 in MDA‐MB‐231 and MDA‐MB‐468 cells. B–E) The capacity of shSC and shNUSAP1 MDA‐MB‐231 cells in migration (B,C) and invasion (D,E) was examined by Boyden chamber assays. Scale bar, 60 µm. F,G) Colony formation assays were performed to evaluate the survival of shSC and shNUSAP1 MDA‐MB‐231 cells. H,I) Growth curve (H) and tumor weights (I) of shSC and shNUSAP1#1 MDA‐MB‐468 tumors. n = 5 mice per group. J–L) Representative images of IHC staining or H&E staining of the indicated xenograft tumors using the indicated antibodies (J). Magnified images of the boxed area are shown in the insets. Scale bar, 40 µm. Quantification of cleaved caspase 3 (CC3)‐positive cell numbers (K) and Ki‐67‐positive cell numbers (L) in tumors. n = 5 mice per group. M,N) Representative images of H&E staining of the lung in the mice bearing indicated xenograft tumors (M). Scale bar, 100 µm. Lung metastasis burden was determined by qPCR (N). n = 5 mice per group. O) Kaplan–Meier curves for overall survival in TNBC patients with low (n = 6) or high (n = 13) NUSAP1 mRNA levels in the PrognoScan database. P,Q) The protein levels of NUSAP1 in paired TNBC tissues and adjacent normal breast tissues were determined by western blotting (n = 10). P, patient; N, normal breast tissue; T, TNBC tissue. ^***^ *P* < 0.001, ^****^ *P* < 0.0001, by 2‐way ANOVA Tukey's multiple comparisons test (C,E,G,H) or unpaired t test (two‐tailed) (I,K,L,N,Q).

NUSAP1 exhibits context‐dependent roles in cancer, with its expression significantly reduced in invasive breast carcinomas compared to ductal carcinoma in situ,^[^ ^23^ ^]^ indicative of its distinct roles across breast cancer subtypes. To determine the precise role of NUSAP1 in invasive breast carcinomas, we established NUSAP1 knockdown (KD) cell lines using two independent shRNA targeting *NUSAP1* in TNBC cells, which present the most aggressive breast cancer subtype and lack effective therapeutic targets (**Figure** **1**A). NUSAP1 ablation significantly promoted the proliferation of MDA‐MB‐231 and MDA‐MB‐468 cells (Figure S1A,B, Supporting Information). Since cancer mortality is predominantly caused by metastasis, we assessed the influence of NUSAP1 on TNBC cell migration and invasion using Boyden chamber assays. Compared with the scrambled control (shSC) MDA‐MB‐231 cells, NUSAP1 knockdown in MDA‐MB‐231 cells resulted in an increased capacity for migration and invasion under both normoxic and hypoxic conditions (Figure 1B–E). Similar results were observed for MDA‐MB‐468 cells (Figure S1C–F, Supporting Information). Taken together, these data suggest that NUSAP1 inhibits TNBC cell migration and invasion. Furthermore, NUSAP1 depletion increased the colony numbers of MDA‐MB‐231 and MDA‐MB‐468 cells, as shown by the clonogenic assays (Figure 1F,G; Figure S1G,H, Supporting Information), indicating that NUSAP1 suppresses TNBC cell survival. In summary, NUSAP1 functions as a tumor suppressor in TNBC cells in vitro.

To determine whether NUSAP1 inhibits TNBC growth and metastasis in vivo, shSC or shNUSAP1#1 MDA‐MB‐468 cells were orthotopically implanted into the mammary fat pads of female non‐obese diabetic/severe combined immunodeficiency (NOD‐SCID) mice. NUSAP1 ablation significantly augmented MDA‐MB‐468 tumor growth (Figure 1H,I; Figure S1I, Supporting Information). Moreover, we performed immunohistochemistry (IHC) staining assays and found that NUSAP1 depletion dramatically reduced the protein levels of cleaved caspase‐3 (CC3), a widely used marker of cell death (Figure 1J,K). In contrast, the protein level of Ki‐67, a well‐known cell proliferation marker, was remarkably increased by NUSAP1 knockdown (Figure 1J,L). H&E staining showed that NUSAP1 ablation robustly enhanced the lung metastasis of MDA‐MB‐468 cells (Figure 1M,N). Collectively, NUSAP1 is required for TNBC growth and lung metastasis in mice.

Furthermore, we analyzed the correlation between NUSAP1 mRNA levels and survival of TNBC patients and found that NUSAP1 mRNA levels were positively correlated with overall survival of TNBC patients (Figure 1O), as evidenced by Kaplan‐Meier analysis of the PrognoScan database (https://dna00.bio.kyutech.ac.jp/PrognoScan/index.html).^[^ ^24^ ^]^ Consistently, the protein levels of NUSAP1 were markedly lower in TNBC tissues than in the adjacent normal breast tissues (Figure 1P,Q). Taken together, decreased NUSAP1 expression is correlated with poor clinical outcomes in patients with breast cancer.

### 2.2 NUSAP1 Interacts with HIFα and Inhibits HIF Transcriptional Activity

**Figure 2:**
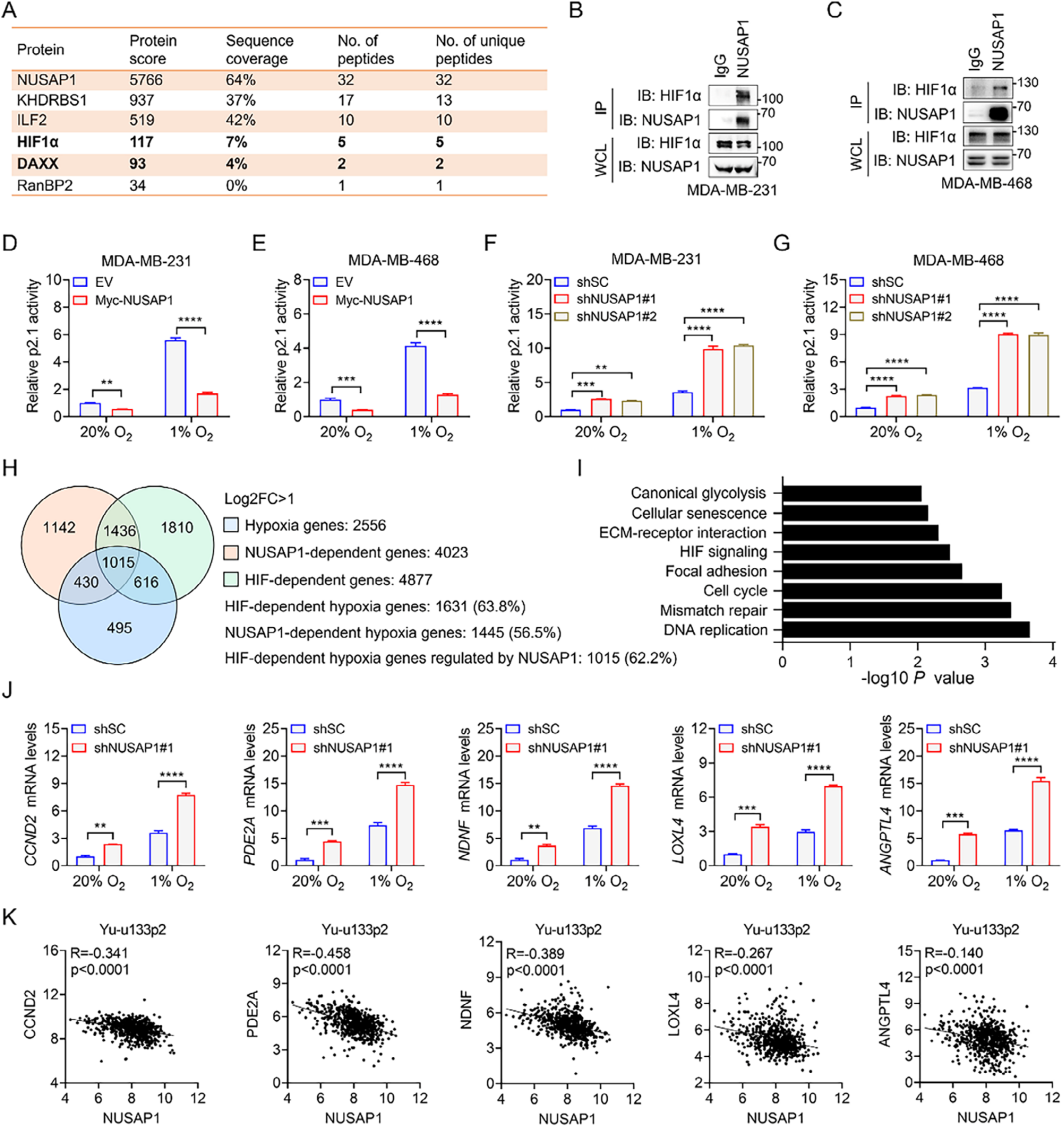
NUSAP1 is a corepressor of HIF1α. A) Representatives of potential NUSAP1‐interacting proteins identified by liquid chromatography‐tandem mass spectrometry (LC‐MS/MS). B,C) Co‐immunoprecipitation (Co‐IP) and immunoblotting (IB) analyses were conducted in MDA‐MB‐231 (B) and MDA‐MB‐468 (C) cells treated with MG132 (10 µM) for 6 h to stabilize HIF1α protein, using the indicated antibodies. D–G) Luciferase reporter assays in MDA‐MB‐231 and MDA‐MB‐468 cells with NUSAP1 overexpression (D,E) or ablation (F,G). H) Venn diagram of NUSAP1 and HIF target genes in MDA‐MB‐231 cells exposed to 20% or 1% O_2_ for 24 h (n = 2). I) KEGG (Kyoto Encyclopedia of Genes and Genomes) analysis of NUSAP1‐dependent HIF target genes (n = 2). J) The mRNA levels of indicated NUSAP1‐dependent HIF target genes were determined in shSC and shNUSAP1#1 MDA‐MB‐231 cells exposed to 20% or 1% O_2_ for 24 h. K) The correlation of mRNA levels of NUSAP1 and indicated HIF target genes in human breast tumors and normal breast tissues. ^**^ *P* < 0.01, ^***^ *P* < 0.001, ^****^ *P* < 0.0001, by 2‐way ANOVA Tukey's multiple comparisons test (D–G,J) or Pearson correlation test (two‐tailed) (K).

To elucidate the mechanism by which NUSAP1 suppresses TNBC growth and metastasis, MDA‐MB‐231 cells stably expressing Flag‐NUSAP1 were subjected to liquid chromatography‐tandem mass spectrometry (LC‐MS/MS) to identify potential NUSAP1‐binding proteins. LC‐MS/MS analysis revealed HIF1α to be a high‐confidence putative NUSAP1‐interacting protein (**Figure** **2**A). Notably, RanBP2 and ILF2, two well‐known NUSAP1‐interacting proteins, were also identified, thus validating our experimental approach (Figure 2A). Endogenous NUSAP1‐HIF1α interaction was confirmed in multiple TNBC cell lines, including MDA‐MB‐231, MDA‐MB‐468, and HCC1937 (Figure 2B,C; Figure S2A–D, Supporting Information). To map the interaction domains, we performed domain‐mapping assays, which demonstrated that HIF1α binds to the 158–410 amino acid (aa) region of NUSAP1 via its oxygen‐dependent degradation domain (ODD) (Figure S2E–H, Supporting Information). Because the microtubule‐associated domain (MAD) resides within the aa 158–410 region (Figure S2F, Supporting Information), we further investigated ODD‐MAD interaction. GST pull‐down assays confirmed that NUSAP1 associates with the ODD of HIF1α through its MAD (Figure S2I, Supporting Information). Notably, NUSAP1 was also associated with HIF2α, a HIF1α paralog, in TNBC cells (Figure S2J–L, Supporting Information). In conclusion, NUSAP1 interacted with both HIF1α and HIF2α in human TNBC cells.

HIFα is a crucial subunit of the HIF transcription factor. Therefore, we hypothesized that NUSAP1 regulates HIF transcriptional activity. To test this hypothesis, we performed HIF luciferase reporter assays using p2.1, a widely used HIF luciferase reporter vector containing 2 × HREs from human *VEGFA*, a shared target gene of HIF1 and HIF2.^[^ ^25^, ^26^ ^]^ NUSAP1 overexpression significantly inhibited HIF reporter activity in MDA‐MB‐231 and MDA‐MB‐468 cells exposed to 20% or 1% O_2_ for 24 h (Figure 2D,E), whereas NUSAP1 depletion dramatically enhanced HIF luciferase reporter activity in TNBC cells (Figure 2F,G). Notably, immunofluorescence staining revealed predominant nuclear localization of NUSAP1 and colocalization with both HIF1α and HIF2α (Figure S2M, Supporting Information) but did not alter the protein levels or subcellular localization of HIF1α and HIF2α (Figure S2M,N, Supporting Information). These results indicated that NUSAP1 restricts HIF transcriptional activity without perturbing its expression or subcellular distribution.

To globally assess NUSAP1's impact on the HIF transcriptome, we performed RNA‐sequencing (RNA‐seq) in parental, single cell‐derived clone of HIF1α and HIF2α double knockout (HIF‐DKO, clone #30), and shNUSAP1#1 MDA‐MB‐231 cells cultured in 20% or 1% O_2_ for 24 h. Among 2556 hypoxia‐induced genes, 1631 (63.8%) were HIF‐dependent and 1445 (56.5%) were NUSAP1‐regulated (Figure 2H). Strikingly, NUSAP1 controlled 1015 (62.2%) of the HIF‐dependent hypoxia‐responsive genes (Figure 2H), which were enriched in HIF‐associated processes, including proliferation and metastasis (Figure 2I). Quantitative reverse transcription–polymerase chain reaction (RT‐qPCR) validation demonstrated that NUSAP1 depletion significantly increased the transcription of HIF target genes involved in tumor growth (*CCND2* and *PDE2A*) and metastasis (*NDNF*, *LOXL4*, and *ANGPTL4*) in MDA‐MB‐231 cells (Figure 2J), while NUSAP1 overexpression attenuated their transcription in MDA‐MB‐468 cells (Figure S2O, Supporting Information). Importantly, NUSAP1 mRNA levels were inversely correlated with those of these HIF target genes in human breast cancer tissues (Figure 2K). Taken together, NUSAP1 suppressed HIF transcriptional activity and downstream target gene expression in TNBC cells.

### 2.3 NUSAP1 Suppresses TNBC cells’ Oncogenic Potential by Inhibiting HIF In Vitro and In Vivo

**Figure 3:**
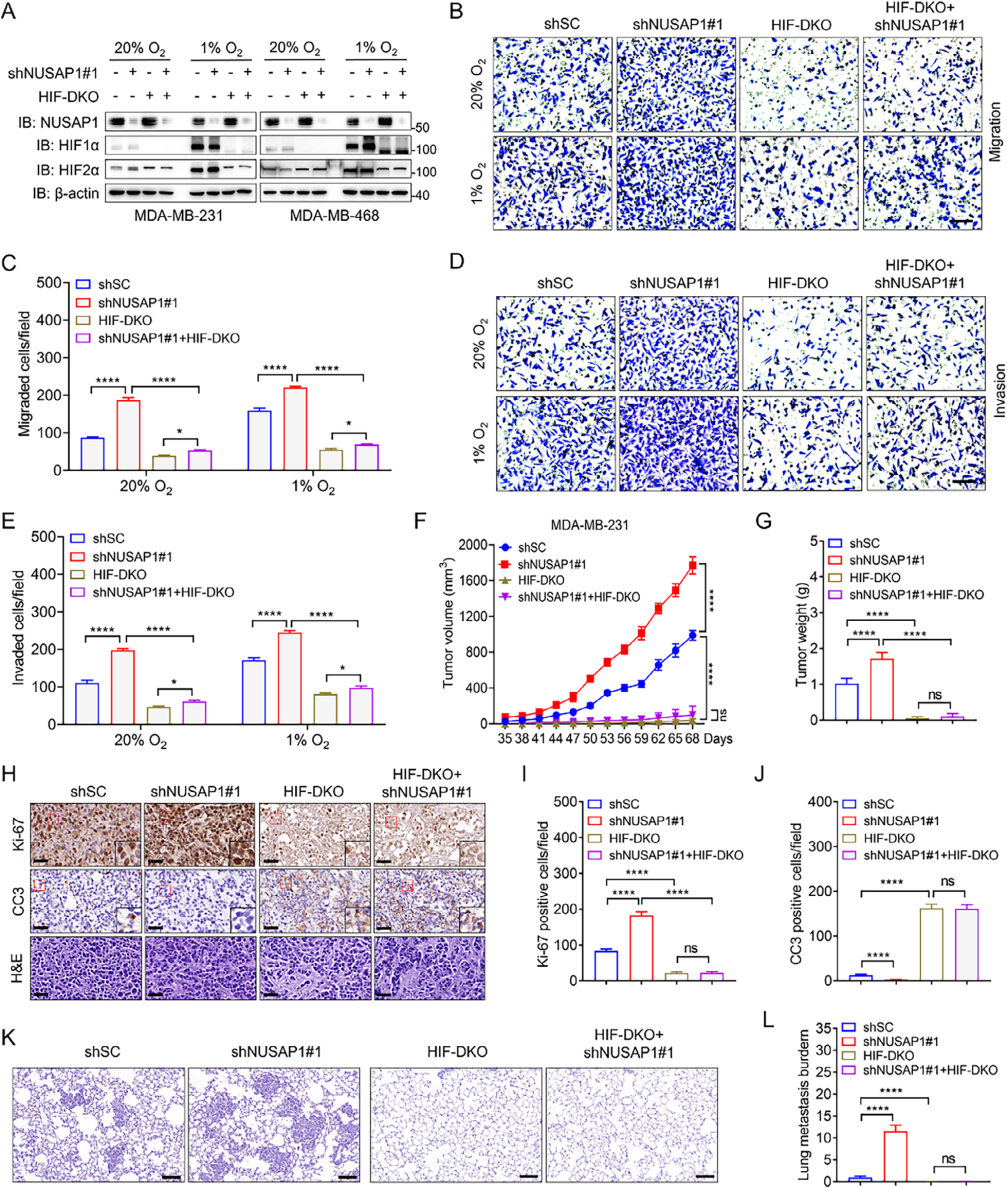
NUSAP1 restricts HIF‐mediated oncogenic potential of TNBC cells in vitro and in vivo. A) NUSAP1 was depleted in HIF‐DKO MDA‐MB‐231 (clone #30) and HIF‐DKO MDA‐MB‐468 (clone #5) cell lines, respectively. Protein levels of HIF1α, HIF2α, and NUSAP1 were examined in the indicated MDA‐MB‐231 and MDA‐MB‐468 cell lines exposed to 20% or 1% O2 for 6 h. B–E) The capacity of indicated MDA‐MB‐231 cells in migration (B,C) and invasion (D,E) was examined by Boyden chamber assays. Scale bar, 60 µm. F,G) Growth curve (F) and tumor weights (G) of indicated xenograft tumors. n = 5 mice per group. H–J) Representative images of IHC staining or H&E staining of the indicated xenograft tumors using the indicated antibodies (H). Magnified images of the boxed area are shown in the insets. Scale bar, 40 µm. Quantification of Ki‐67‐positive cell numbers (I) and CC3‐positive cell numbers (J) in tumors. n = 5 mice per group. K,L) Representative images of H&E staining of the lung in the mice bearing indicated xenograft tumors (K). Scale bar, 100 µm. Lung metastasis burden was determined by qPCR (L). n = 5 mice per group. ^*^ *P* < 0.05, ^****^ *P* < 0.0001, by 2‐way ANOVA Tukey's multiple comparisons test (C,E,F) or 1‐way ANOVA Tukey's multiple comparisons test (G,I,J,L). ns, no significance.

Based on these findings, we investigated whether NUSAP1 exerts its tumor‐suppressive effects through HIF in TNBC cells. To test this hypothesis, NUSAP1 was depleted in HIF‐DKO (clone #30) MDA‐MB‐231 and HIF‐DKO (clone #5) MDA‐MB‐468 cells to assess the mRNA levels of *ANGPTL4* and cell proliferation, migration, and invasion capabilities in vitro (**Figure** **3**A). As expected, NUSAP1 ablation enhanced *ANGPTL4* transcription and the proliferation, migration, and invasion of MDA‐MB‐231 cells, and these effects were abolished by HIF‐DKO (Figure 3B–E; Figure S3A,B, Supporting Information). Similar results were observed for MDA‐MB‐468 cells (Figure S3C–G, Supporting Information), confirming that NUSAP1 suppresses the oncogenic properties of TNBC cells by inhibiting HIF in vitro. To validate this conclusion in vivo, we performed xenograft assays in female NOD‐SCID mice by implanting parental, shNUSAP1#1, HIF‐DKO, and shNUSAP1#1 + HIF‐DKO MDA‐MB‐231 cells into mammary fat pads. The shNUSAP1#1‐induced increase in tumor growth was completely reversed by HIF‐DKO (Figure 3F,G; Figure S3H, Supporting Information). IHC analysis demonstrated that shNUSAP1#1‐enhanced cell proliferation was abrogated by HIF‐DKO as evidenced by Ki‐67 staining (Figure 3H,I). In contrast, CC3 staining indicated that the decreased cell death induced by shNUSAP1#1 was restored by HIF‐DKO (Figure 3H,J). Furthermore, shNUSAP1#1‐promoted lung metastasis was abolished by HIF‐DKO (Figure 3K,L). The efficiency of shNUSAP1#1 and HIF‐DKO in tumors was verified by western blotting (Figure S3I, Supporting Information). Collectively, these data demonstrated that NUSAP1 inhibits TNBC growth and metastasis by suppressing HIF in vivo.

Notably, NUSAP1 possesses HIF‐independent functions, because NUSAP1 depletion‐mediated phenotypes were not fully restored by HIF‐DKO. Previous studies show that NUSAP1 may regulate p53 or Wnt signaling pathway,^[^ ^5^, ^27^ ^]^ prompting us to determine whether NUSAP1 modulates the expression of their canonical target genes. RT‐qPCR analysis indicated that NUSAP1 knockdown downregulated the expression of p53 target genes in both normoxic and hypoxic MDA‐MB‐231 cells, including *BBC3* (encoding PUMA) and *PMAIP1* (encoding NOXA), which encode tumor suppressors (Figure S3J,K, Supporting Information). Intriguingly, p53 target genes are not regulated by HIF or hypoxia,^[^ ^28^, ^29^ ^]^ ruling out the possibility that NUSAP1 controls p53 target genes through HIF. On the contrary, NUSAP1 ablation upregulated the mRNA levels of *Vimentin* and *PPARD* (Figure S3L,M, Supporting Information), which are Wnt target genes. Notably, HIF indirectly activates the Wnt signaling pathway in MDA‐MB‐231 cells.^[^ ^30^ ^]^ Hence, we cannot exclude the possibility that NUSAP1 negatively regulates the Wnt signaling pathway by repressing HIF. Nevertheless, NUSAP1 possesses HIF‐independent functions, at least partially through p53, to control TNBC proliferation.

### 2.4 NUSAP1 Inhibits HIF Transcriptional Activity by Recruiting DAXX

**Figure 4:**
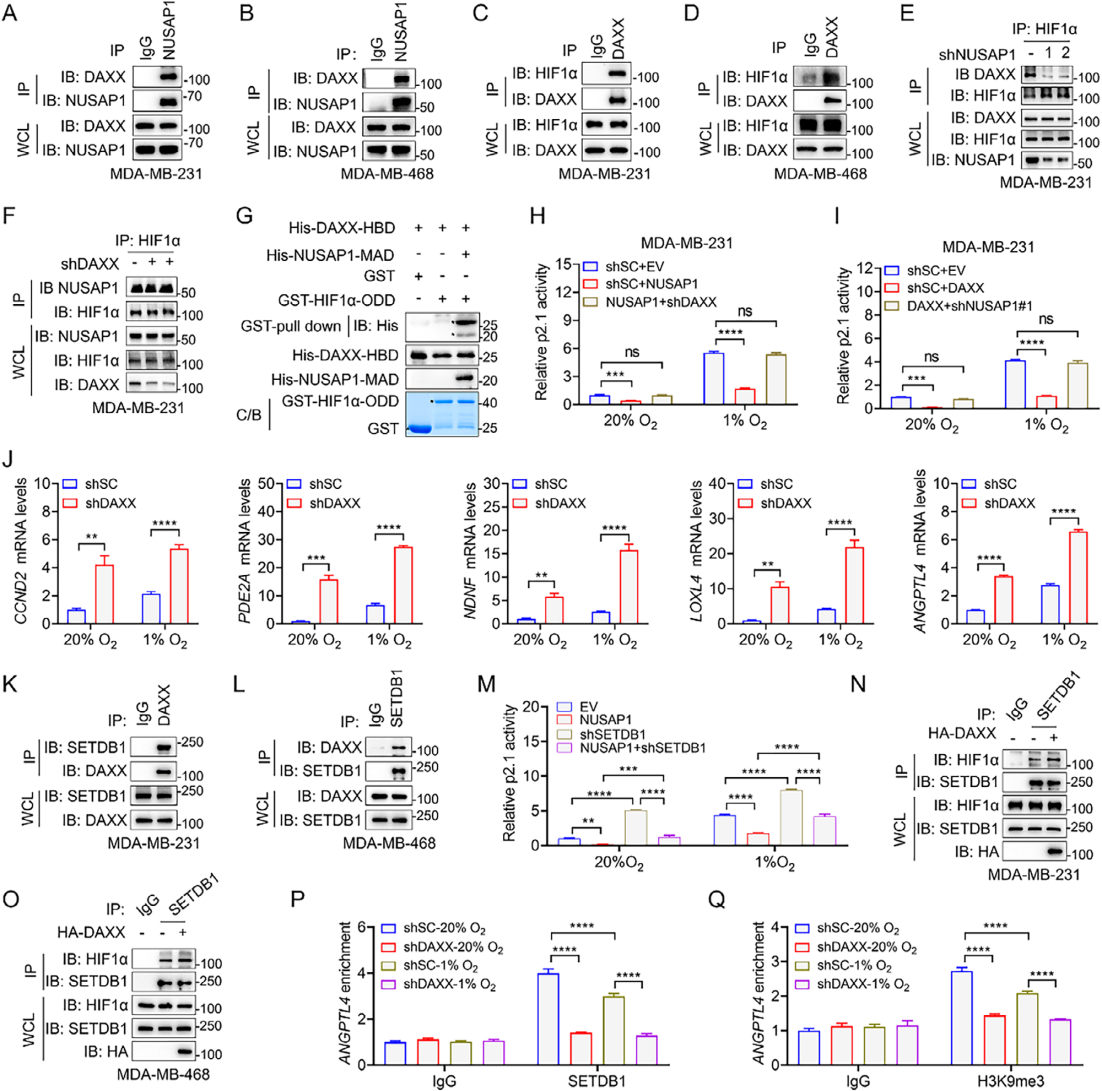
NUSAP1 inhibits HIF transcriptional activity by recruiting DAXX. A–F) Co‐IP and IB analyses were conducted using the indicated antibodies in MDA‐MB‐231 and MDA‐MB‐468 cells cultured in 1% O_2_ for 6 h. G) Pull‐down and IB analyses were performed using the indicated antibodies and purified proteins in vitro. C/B, Coomassie blue staining; HBD, histone‐binding domain; ODD, oxygen‐dependent degradation domain. H,I) Luciferase reporter assays in MDA‐MB‐231 cells transfected with indicated constructs and exposed to 20% or 1% O_2_ for 24 h. J) The mRNA levels of indicated HIF target genes were determined by RT‐qPCR in shSC and shDAXX MDA‐MB‐231 cells exposed to 20% or 1% O_2_ for 24 h. K,L) Co‐IP and IB analyses were conducted using the indicated antibodies in normoxic MDA‐MB‐231 and MDA‐MB‐468 cells. M) Luciferase reporter assays in the indicated MDA‐MB‐231 cell lines exposed to 20% O_2_ or 1% O_2_ for 24 h. N,O) Co‐IP and IB analyses were conducted using the indicated antibodies in MDA‐MB‐231 and MDA‐MB‐468 cells exposed to 20% O_2_ or 1% O_2_ for 6 h. P,Q) Chromatin immunoprecipitation (ChIP) assays were performed in the indicated MDA‐MB‐231 cell lines exposed to 20% or 1% O_2_ for 24 h using the indicated antibodies, followed by qPCR analysis. ^**^ *P* < 0.01, ^***^ *P* < 0.001, ^****^ *P* < 0.0001, by 2‐way ANOVA Tukey's multiple comparisons test (H‐J,M,P,Q). ns, no significance.

To mechanistically define how NUSAP1 suppresses HIF transcriptional activity, we re‐analyzed our LC‐MS/MS data and identified two transcriptional corepressors, DAXX and KHDRBS1, as potential NUSAP1 interactors (Figure 2A). NUSAP1 bound to both proteins (Figure S4A,B, Supporting Information), only DAXX remarkably attenuated the HIF transcriptional activity in HEK293T cells (Figure S4C,D, Supporting Information). While DAXX is known to be hypoxia‐repressed via HIF during Slug‐HDAC1 complex‐mediated epithelial–mesenchymal transition (EMT) and invasion,^[^ ^17^, ^31^ ^]^ its regulation of HIF remains unexplored. We hypothesized that NUSAP1 inhibits HIF transcriptional activity through DAXX. To confirm this hypothesis, we examined DAXX‐NUSAP1 and DAXX‐HIF1α interactions in TNBC cells. As expected, DAXX interacted with both HIF1α and NUSAP1 in MDA‐MB‐231 and MDA‐MB‐468 cells (**Figure** **4**A–D; Figure S4E–H, Supporting Information). Notably, hypoxia showed only mild suppression of DAXX protein levels in these TNBC cells (Figure S4I, Supporting Information) compared to lung cancer cells.^[^ ^17^ ^]^ Domain mapping assays revealed that DAXX binds NUSAP1 MAD and HIF1α ODD through its histone‐binding domain (HBD) (Figure S4J–N, Supporting Information). Notably, NUSAP1 ablation substantially weakened DAXX‐HIF1α binding, whereas DAXX depletion did not affect the NUSAP1‐HIF1α association in MDA‐MB‐231 and MDA‐MB‐468 cells (Figure 4E,F; Figure S4O, Supporting Information). Conversely, the overexpression of NUSAP1 strengthened the DAXX‐HIF1α association (Figure S4P, Supporting Information). These findings suggest that NUSAP1 is indispensable for the DAXX‐HIF1α interaction in TNBC cells. To further explore whether NUSAP1 bridges DAXX and HIF1α through its MAD, we conducted GST pull‐down assays and demonstrated that His‐NUSAP1‐MAD was required for GST‐HIF1α‐ODD/His‐DAXX‐HBD complex formation (Figure 4G). Together, these data establish that NUSAP1 scaffolds the DAXX‐HIF1α interaction via its MAD, revealing a novel mechanism of HIF regulation in TNBC cells.

To establish a functional relationship between NUSAP1 and DAXX in HIF regulation, we performed HRE luciferase reporter assays in MDA‐MB‐231 cells. Although NUSAP1 overexpression suppressed HIF activity, this inhibition was completely reversed by DAXX knockdown (Figure 4H). Reciprocally, NUSAP1 depletion abolished the DAXX‐mediated repression of HIF transcriptional activity in MDA‐MB‐231 cells (Figure 4I), demonstrating their mutual dependence on HIF regulation. To further support this conclusion, the mRNA levels of NUSAP1‐regulated HIF target genes, including *CCND2*, *PDE2A*, *NDNF*, *LOXL4*, and *ANGPTL4*, were assessed using RT‐qPCR in MDA‐MB‐231 cells with DAXX depletion. As expected, DAXX ablation significantly upregulated the mRNA levels of these genes in the MDA‐MB‐231 cells (Figure 4J). In accordance with these results, clinical correlation analysis revealed an inverse relationship between DAXX and HIF target genes in breast tumors (Figure S4Q, Supporting Information), underscoring the pathophysiological relevance of this regulatory axis. Moreover, HIF‐DKO abolished shDAXX‐induced upregulation of *ANGPTL4* in MDA‐MB‐231 cells (Figure S4R, Supporting Information). Collectively, these results established that NUSAP1 is a critical scaffolding protein that orchestrates the assembly of the HIF‐DAXX repressive complex, thereby suppressing HIF‐mediated transcriptional activation in TNBC cells.

DAXX usually recruits methylatransferase SETDB1 or SUV39H1 to catalyze the trimethylation of histone 3 at lysine 9 (H3K9me3), thereby inhibiting gene transcription.^[^ ^32^, ^33^ ^]^ Therefore, we attempted to investigate whether DAXX abolishes HIF transcription activity by modulating H3K9me3. DAXX interacted with both SETDB1 and SUV39H1 in MDA‐MB‐231 and MDA‐MB‐468 cells (Figure 4K,L; Figure S4S, Supporting Information). HRE‐luciferase reporter assays indicated that NUSAP1‐mediated HIF transcriptional activity repression was rescued by SETDB1 ablation, but not by SUV39H1 depletion (Figure 4M; Figure S4T, Supporting Information). Additionally, DAXX overexpression increased the HIF1α‐SETDB1 interaction in MDA‐MB‐231 and MDA‐MB‐468 cells (Figure 4N,O). Furthermore, chromatin immunoprecipitation (ChIP)‐qPCR analysis indicated that SETDB1 and H3K9me3 were enriched in the HRE region of the HIF target gene *ANGPTL4*, and this enrichment was abolished by DAXX ablation in MDA‐MB‐231 cells (Figure 4P,Q). Taken together, DAXX recruits SETDB1 to elevate the H3K9me3 levels in the HRE region of HIF target genes, thereby repressing HIF transcriptional activity.

### 2.5 NUSAP1 and DAXX Synergistically Suppress TNBC Oncogenicity

Our finding that NUSAP1 inhibits HIF via DAXX recruitment prompted us to investigate its functional interdependence in TNBC cells. NUSAP1 overexpression significantly attenuated MDA‐MB‐231 cell invasion, and this effect was largely reversed by DAXX knockdown (Figure S5A–C, Supporting Information). Conversely, the DAXX‐mediated suppression of MDA‐MB‐231 cell invasion was robustly rescued by NUSAP1 depletion (Figure S5D–F, Supporting Information), demonstrating reciprocal functional dependence. Notably, the oncogenic phenotypes observed in NUSAP1 + shDAXX and DAXX + shNUSAP1#1 MDA‐MB‐231 cells were less pronounced than those observed in the single knockdown control (Figure S5B,C,E,F, Supporting Information), suggesting that DAXX and NUSAP1 have shared and distinct tumor‐suppressive functions. Collectively, these findings demonstrated that DAXX and NUSAP1 act as cooperative tumor suppressors in TNBC cells, and their functional synergy is mediated, at least in part, through physical complex formation.

**Figure 5:**
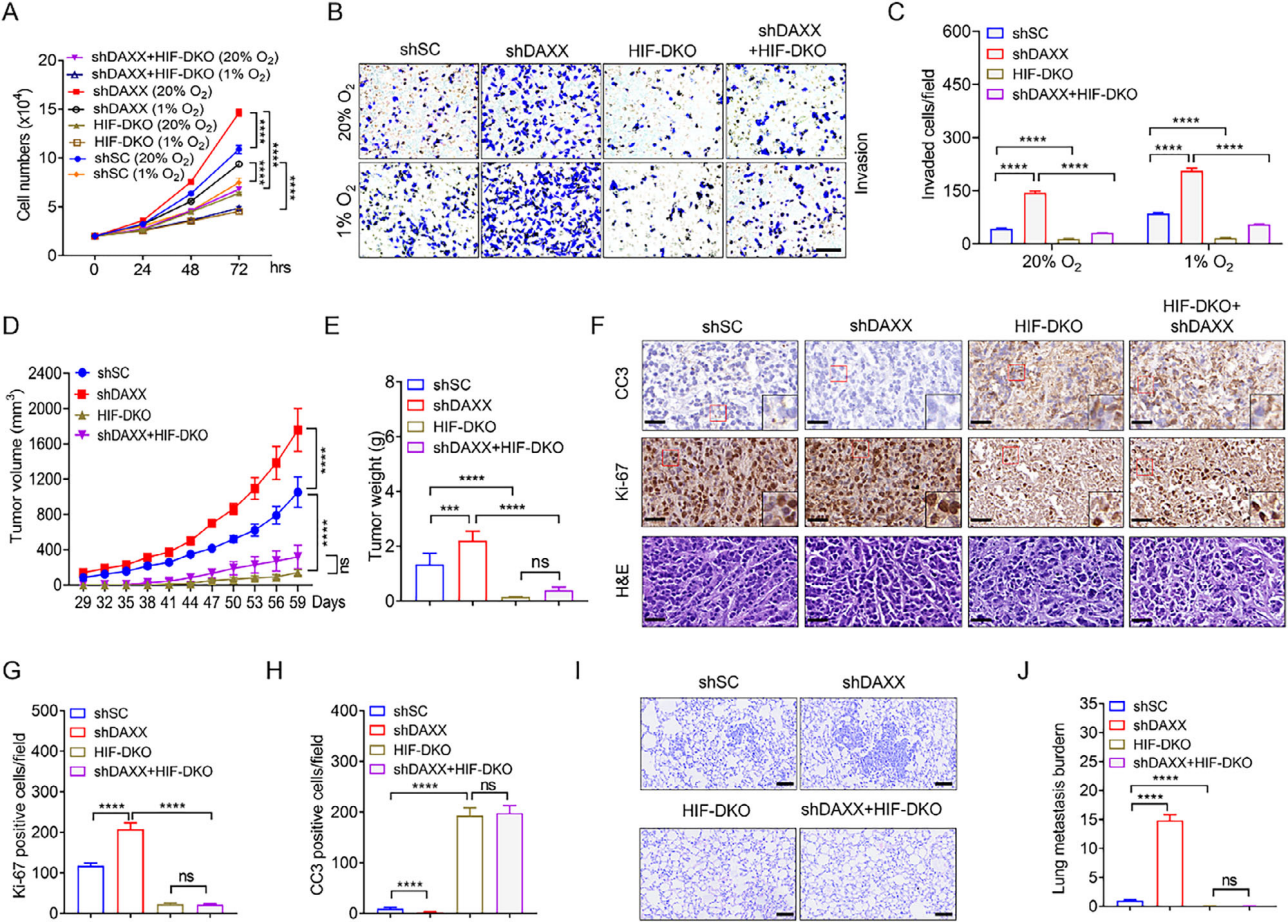
DAXX inhibits HIF‐mediated TNBC progression. A) The proliferation of indicated MDA‐MB‐231 cells exposed to 20% or 1% O_2_ for 24, 48 or 72 h. B,C) The capacity of indicated MDA‐MB‐231 cells in invasion (B,C) was detected by Boyden chamber assays. Scale bar, 60 µm. D,E) Growth curve (D) and tumor weights (E) of indicated xenograft tumors. n = 5 mice per group. F–H) Representative images of IHC staining or H&E staining of the indicated xenograft tumors using the indicated antibodies (F). Magnified images of the boxed area are shown in the insets. Scale bar, 40 µm. Quantification of Ki‐67‐positive cell numbers (G) and CC3‐positive cell numbers (H) in tumors. n = 5 mice per group. I,J) Representative images of H&E staining of the lung in the mice bearing indicated xenograft tumors (I). Scale bar, 100 µm. Lung metastasis burden was determined by qPCR (J). n = 5 mice per group. ^***^ *P* < 0.001, ^****^ *P* < 0.0001, by 2‐way ANOVA Tukey's multiple comparisons test (A,C,D) or 1‐way ANOVA Tukey's multiple comparisons test (E,G,H,J). ns, no significance.

To determine the inhibitory effect of DAXX on HIF in vitro and in vivo, DAXX was depleted in HIF‐DKO (clone #30) MDA‐MB‐231 or HIF‐DKO (clone #5) MDA‐MB‐468 cells (Figure S5G,H, Supporting Information). Cell proliferation and invasion assays showed that HIF‐DKO completely reversed the enhanced proliferation and invasion resulting from DAXX ablation (**Figure** **5**A–C; Figure S5I,J, Supporting Information). The phenotypic rescue by HIF‐DKO was more pronounced than that in shDAXX + HIF‐DKO cells (Figure 5A–C; Figure S5I,J, Supporting Information), suggesting that HIF is the primary downstream effector of DAXX, whereas DAXX retains additional HIF‐independent functions. Next, we conducted xenograft assays by injecting shSC, shDAXX, HIF‐DKO, or shDAXX + HIF‐DKO MDA‐MB‐231 cells into the fat pads of female NOD‐SCID mice. As expected, DAXX knockdown promoted tumor growth, whereas HIF‐DKO counteracted this effect (Figure 5D,E; Figure S5K,L, Supporting Information). IHC and H&E staining indicated that HIF‐DKO blocked the DAXX ablation‐induced increase in cell proliferation and lung metastasis, while restoring the diminished cell death observed in DAXX‐depleted tumors (Figure 5F–J). These results demonstrate DAXX‐mediated repression of HIF‐driven oncogenesis in TNBC.

### 2.6 TS‐MAD, a NUSAP1‐Derived Mini‐Protein, Inhibits HIF Transcriptional Activity and TNBC Progression

The MAD of NUSAP1 was necessary and sufficient for the DAXX‐HIF1α interaction (Figure 4G), and NUSAP1 exerted its tumor suppressor functions in a nuclear localization‐dependent manner. To harness these mechanisms for anticancer therapy, we generated constructs expressing Flag‐tagged MAD containing amino acid 186–211 (the nuclear localization sequence, NLS) of NUSAP1 (referred to as NN‐MAD).^[^ ^2^ ^]^ Flag‐tagged MAD without an NLS (referred to as MAD) served as the negative control (Figure S6A, Supporting Information). Immunofluorescence assays indicated that MAD predominantly localized to the cytosol but exerted no effect on microtubules (Figure S6B, Supporting Information). In contrast, NN‐MAD was primarily localized to the cytosol and induced microtubule bundling (Figure S6B, Supporting Information). Therefore, we generated another MAD construct using a canonical simian virus 40 (SV40) NLS (PKKKRKV) (Figure S6A, Supporting Information), which predominantly accumulated in the nucleus while maintaining a normal microtubule architecture (Figure S6B, Supporting Information). Importantly, luciferase reporter assays showed that MAD with the SV40 NLS dramatically inhibited HIF transcriptional activity, whereas NN‐MAD had only a mild effect (Figure S6C, Supporting Information). Thus, we designated Flag‐tagged MAD with the SV40 NLS as Tumor Suppressor‐MAD (TS‐MAD) and generated TS‐MAD‐expressing MDA‐MB‐231 and MDA‐MB‐468 cell lines (Figure S6A,D, Supporting Information).

**Figure 6:**
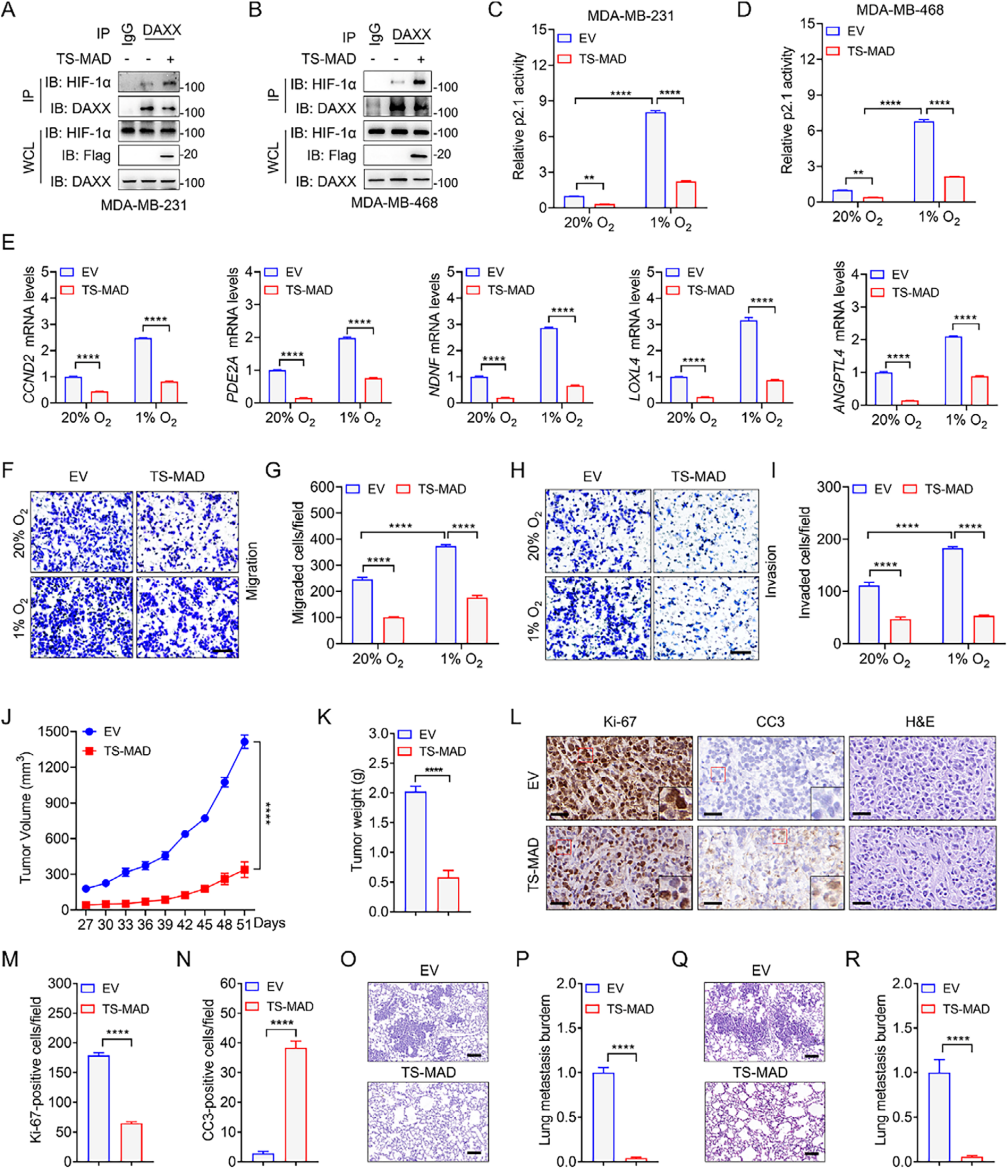
TS‐MAD recaptures NUSAP1's tumor‐suppressive effects in TNBC. A,B) MDA‐MB‐231 (A) and MDA‐MB‐468 (B) cells with EV or vector encoding TS‐MAD were treated with MG132 for 6 h before harvest and then subjected to co‐IP and IB analyses using the indicated antibodies. C,D) Luciferase reporter assays in MDA‐MB‐231 (C) and MDA‐MB‐468 (D) cells with or without TS‐MAD and exposed to 20% or 1% O_2_ for 24 h. E) The mRNA levels of indicated HIF target genes were determined by RT‐qPCR in EV and TS‐MAD MDA‐MB‐231 cells exposed to 20% or 1% O_2_ for 24 h. F‐I) The migration (F,G) and invasion (H,I) abilities of indicated MDA‐MB‐231 cells were determined by Boyden chamber assays. Scale bar, 60 µm. J,K) Growth curve (J) and tumor weights (K) of indicated xenograft tumors. n = 5 mice per group. L‐N) Representative images of IHC staining of the indicated xenograft tumors using the indicated antibodies (L). Magnified images of the boxed area are shown in the insets. Scale bar, 40 µm. Quantification of Ki‐67‐positive cell numbers (M) and CC3‐positive cell numbers (N) in tumors. n = 5 mice per group. O,P) Representative images of H&E staining of the lung in the mice bearing indicated xenograft tumors (O). Scale bar, 100 µm. Lung metastasis burden was determined by qPCR (P). n = 5 mice per group. Q,R) Representative images of H&E staining of the lung in the mice injected with EV and TS‐MAD MDA‐MB‐231 cells via tail vein (Q). Scale bar, 100 µm. Lung metastasis burden was determined by qPCR (R). n = 5 mice per group. ^**^ *P* < 0.01, ^****^ *P* < 0.0001, by 2‐way ANOVA Tukey's multiple comparisons test (C,D,E,G,I,J) or unpaired t test (two‐tailed) (K,M,N,P,R).

In line with our hypothesis, TS‐MAD expression enhanced the DAXX‐HIF1α interaction and suppressed p2.1 luciferase reporter activity in MDA‐MB‐231 and MDA‐MB‐468 cells (**Figure** **6**A–D). Furthermore, TS‐MAD dramatically reduced the transcription of HIF target genes, including *CCND2*, *PDE2A*, *NDNF*, *LOXL4*, and *ANGPTL4* in MDA‐MB‐231 and MDA‐MB‐468 cells (Figure 6E; Figure S6E, Supporting Information), demonstrating a functional similarity to full‐length NUSAP1 in blocking HIF transcriptional activity. We tested the effect of TS‐MAD on TNBC cell proliferation and metastasis. TS‐MAD potently inhibited the proliferation, migration, and invasion of MDA‐MB‐231 and MDA‐MB‐468 cells under both normoxic and hypoxic conditions (Figure 6F–I; Figure S6F–K, Supporting Information). For in vivo validation, MDA‐MB‐231 cells expressing either an empty vector (EV) or TS‐MAD were injected into the mammary fat pads of female NOD‐SCID mice. Mirroring the in vitro results, TS‐MAD markedly suppressed tumor growth, phenocopying the effect of HIF‐DKO (Figure 6J,K; Figure S6L, Supporting Information). TS‐MAD significantly inhibited cell proliferation while triggering cell death in MDA‐MB‐231 tumors, as evidenced by IHC staining using anti‐CC3 and anti‐Ki‐67 antibodies (Figure 6L–N). TS‐MAD also significantly inhibited the lung metastasis of MDA‐MB‐231 cells (Figure 6O,P). To rule out the possibility that the decreased lung metastasis was due to a smaller tumor size, we additionally performed a tail vein injection. In this case, TS‐MAD also significantly mitigated lung metastasis of MDA‐MB‐231 tumor cells in mice (Figure 6Q,R). Importantly, the inhibitory effect of TS‐MAD on MCF10A, a normal breast epithelial cell line, was very weak (Figure S6M,N, Supporting Information). Moreover, TS‐MAD did not alter the chromatin structure of other DAXX‐regulated genes, such as p21 and FASN (Figure S6O,P, Supporting Information), suggesting that TS‐MAD may specifically affect the HIF signaling pathway by recruiting DAXX. Collectively, these results demonstrated that TS‐MAD recapitulates NUSAP1's tumor‐repressive functions, representing a promising therapeutic strategy for HIF‐driven TNBC.

### 2.7 NUSAP1 is a Novel HIF‐Repressed Gene in Breast Cancer Cells

**Figure 7:**
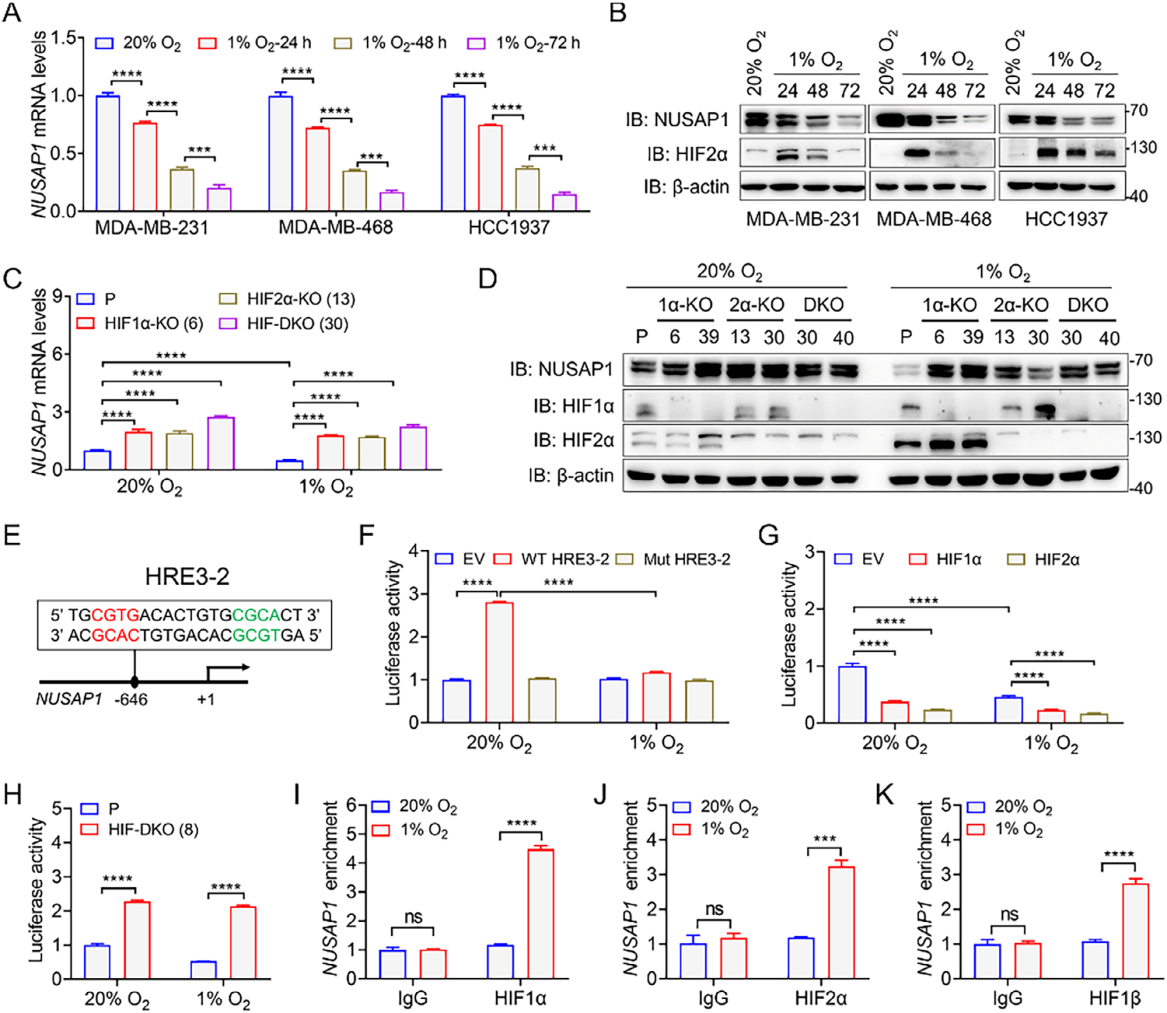
*NUSAP1* is a novel HIF‐repressed gene in TNBC cells. A,B) The mRNA levels (A) and protein levels (B) of NUSAP1 were determined by RT‐qPCR and western blotting, respectively, in MDA‐MB‐231, MDA‐MB‐468, and HCC1937 cells cultured in normoxia (20% O_2_) or hypoxia (1% O_2_) for the indicated times. C) NUSAP1 mRNA levels were quantified by RT‐qPCR in parental (P) MDA‐MB‐231 cells and isogenic single‐cell clones: HIF1α knockout (HIF1α‐KO, clone #6), HIF2α‐KO (clone #13), and HIF1α/HIF2α double KO (HIF‐DKO, clone #30), cultured in 20% O_2_ or 1% O_2_ for 48 h. D) NUSAP1 protein levels were analyzed by western blotting in parental (P) MDA‐MB‐231 cells and isogenic single‐cell clones: HIF1α‐KO (clones #6 and #39), HIF2α‐KO (clones #13 and #30), and HIF‐DKO (clones #30 and #40) maintained in 20% O_2_ or 1% O_2_ for 48 h. E) HRE3‐2 contains a core HRE consensus sequence (in red) as well as an accessorial DNA sequence (in green). F) Luciferase reporter assays in HEK293T cells transfected with empty vector (EV), wild type (WT) HRE3‐2, or mutant (Mut) HRE3‐2 reporter plasmids, together with pSV‐Renilla, followed by exposure to 20% or 1% O_2_ for 24 h. G) Luciferase reporter assays in HeLa cells transfected with EV or vectors encoding HIF1α or HIF2α, along with WT HRE3‐2 and pSV‐Renilla constructs, and exposed to 20% or 1% O_2_ for 24 h. H) Luciferase reporter assays in parental (P) or HIF‐DKO (clone #8) HeLa cells transfected with WT HRE3‐2 and pSV‐Renilla constructs, followed by 20% or 1% O_2_ exposure for 24 h. I–K) ChIP assays were performed in MDA‐MB‐231 cells exposed to 20% or 1% O_2_ for 24 h using the indicated antibodies, followed by qPCR analysis. ^***^ *P* < 0.001, ^****^ *P* < 0.0001, by 2‐way ANOVA Tukey's multiple comparisons test (A,C,F‐K). ns, no significance.

To illustrate the mechanism of NUSAP1 downregulation in TNBC, we re‐analyzed previously published RNA‐seq data^[^ ^34^ ^]^ and expression microarray data.^[^ ^35^ ^]^ These data showed that NUSAP1 mRNA levels were modestly decreased following short‐term hypoxic exposure in MDA‐MB‐231 breast cancer cells (Figure S7A,B, Supporting Information), suggesting that *NUSAP1* is a potential HIF‐repressed gene in TNBC cells. To validate this prediction, we performed RT‐qPCR across multiple breast cancer cell lines exposed to normoxia (20% O_2_) or hypoxia (1% O_2_) for 24, 48, and 72 h. Hypoxia decreased NUSAP1 mRNA levels in a time‐dependent manner in MDA‐MB‐231, MDA‐MB‐468, and HCC1937 cells (**Figure** **7**A). Correspondingly, western blot analysis demonstrated a significant hypoxia‐mediated downregulation of NUSAP1 protein levels in these TNBC cell lines (Figure 7B). Immunofluorescence staining confirmed the predominant nuclear localization of NUSAP1 and its hypoxia‐induced reduction in MDA‐MB‐231 cells cultured in 1% O_2_ for 48 h (Figure S7C, Supporting Information), indicating that sustained hypoxia significantly abrogated NUSAP1 expression. Notably, NUSAP1 mRNA and protein levels were restored in HIF1α‐KO (clones #6 and #39), HIF2α‐KO (clones #13 and #30), and HIF‐DKO (clones #30 and #40) MDA‐MB‐231 cells cultured under both normoxic and hypoxic conditions (Figure 7C,D). Together, these data indicate that hypoxia represses NUSAP1 expression in an HIF‐dependent manner in TNBC cells.

HIFs bind to HREs of downstream target genes to control their transcription.^[^ ^16^ ^]^ To identify functional HREs in the *NUSAP1* promoter, we analyzed the 7‐kb promoter region (from −7000 to −1) of *NUSAP1* using JASPAR, which profiles transcription factor‐binding sites.^[^ ^36^ ^]^ Twelve putative HRE sites were found to be distributed across three subregions: HRE1, HRE2, and HRE3 (Figure S7D, Supporting Information). These subregions were cloned into the pGL3‐basic luciferase reporter plasmid. Luciferase reporter assays showed that reporter gene expression was induced only by HRE3, and this induction was inhibited by hypoxia in HEK293T cells, suggesting that HRE3 is a hypoxia‐responsive subregion (Figure S7E, Supporting Information). We then tested three putative HRE sites within HRE3: HRE3‐1 (−1069 to −1016), HRE3‐2 (−669 to −617), and HRE3‐3 (−448 to −396), by inserting them into the pGL3‐promoter luciferase reporter plasmid for luciferase reporter assays. Only HRE3‐2 was functional and inhibited by hypoxia (Figure 7E; Figure S7F, Supporting Information). Mutation of 5′‐CGTG‐3′ into 5′‐AAAA‐3′ within HRE3‐2 (Mut HRE3‐2) completely abolished its basal activity and hypoxic response in HEK293T cells (Figure 7F). Overexpression of HIF1α or HIF2α dramatically suppressed the wild‐type (WT) HRE3‐2‐driven luciferase activity in both normoxic and hypoxic HeLa cells (Figure 7G). In contrast, the inhibitory effect of hypoxia on HRE3‐2 expression was completely alleviated by HIF‐DKO (clone #8) in HeLa cells (Figure 7H). Therefore, HRE3‐2 is a functional HIF‐dependent hypoxia response element in breast cancer cells. To explore whether *NUSAP1* is a direct HIF target gene in breast cancer cells, chromatin immunoprecipitation‐qPCR (ChIP‐qPCR) was conducted in MDA‐MB‐231 cells exposed to 20% or 1% O_2_ for 24 h. The enrichment of HIF1α and HIF2α in HRE3‐2 was significantly increased by hypoxia in MDA‐MB‐231 cells (Figure 7I,J). Consistent with these results, increased occupancy of HIF1β on HRE3‐2 was also observed in hypoxic MDA‐MB‐231 cells (Figure 7K). Together, these findings indicate that *NUSAP1* is a novel HIF target gene, and its transcription is repressed by HIFs in breast cancer cells.

## 3 Discussion

In this study, we demonstrated that NUSAP1 functions as a scaffold protein that bridges HIF and the transcriptional co‐repressor DAXX through its MAD, thereby impeding HIF transcriptional activity. NUSAP1 depletion enhanced TNBC cell proliferation, migration, and invasion in vitro, and promoted TNBC growth and lung metastasis in vivo. Importantly, we engineered TS‐MAD, which effectively and specifically suppresses HIF transcriptional activity and TNBC progression. Moreover, we identified *NUSAP1* as a novel HIF‐repressing gene in breast cancer cells. These data reveal a negative feedback loop between NUSAP1 and HIF that governs TNBC progression and demonstrate the therapeutic potential of TS‐MAD for TNBC treatment.

HIF1α and HIF2α are highly expressed in most solid tumors and contribute to tumorigenesis and tumor progression by modulating the transcription of several downstream genes. Among these HIF target genes, only a few were repressed by HIF. High‐throughput screening has identified numerous genes that may be repressed by HIF in tumors, but their functional significance and underlying mechanisms in hypoxic microenvironments remain unclear. We demonstrated that *NUSAP1* is a novel HIF‐repressed gene in breast cancer cells, and that this repression exhibits a time‐dependent response to hypoxia, suggesting that the functions of NUSAP1 may be severely impaired due to chronic hypoxia in high‐grade breast tumors. The much lower NUSAP1 mRNA and protein levels observed in TNBC cells are consistent with previous studies demonstrating that HIF1α and HIF2α protein levels are significantly higher in TNBC cells than in other breast cancer subtypes.^[^ ^37^, ^38^ ^]^ The mechanism by which HIFs inhibit NUSAP1 expression requires further investigation. Several epigenetic regulators, such as ZMYND8^[^ ^34^ ^]^ and CHD4^[^ ^39^ ^]^ have been reported to modulate HIF‐mediated transcription. Thus, HIFs may negatively regulate NUSAP1 expression by recruiting epigenetic regulators that modify chromatin structure or DNA methylation. Alternatively, HIF may antagonize other transcription factors that positively regulate NUSAP1 expression. For example, NUSAP1 transcription in HER2+ breast cancer cells is induced by c‐MYC,^[^ ^40^ ^]^, whose transcriptional activity is impeded by HIF through the disruption of c‐MYC binding to MAX or DNA.^[^ ^41^, ^42^ ^]^ Notably, we cannot exclude the possibility that HIF directly inhibits NUSAP1 transcription.

NUSAP1 is a microtubule‐associated protein that regulates mitotic spindle organization, chromosome segregation, and cytokinesis. Emerging evidence suggests that NUSAP1 is involved in cancer biology, although its precise role varies across cancer types, and the underlying mechanisms remain unclear. Analysis of TCGA (The Cancer Genome Atlas) and GEO (Gene Expression Omnibus) datasets revealed increased NUSAP1 expression in many cancers, including breast cancer.^[^ ^43^ ^]^ However, our in vitro and in vivo data convincingly demonstrated that NUSAP1 acts as a tumor suppressor in TNBC by recruiting DAXX to inhibit HIF transcriptional activity. Previous studies have also reported that NUSAP1 exerts tumor‐suppressive effects in breast, colon, and cervical cancers despite its upregulation in these tumors.^[^ ^9^, ^11^ ^]^ Thus, the cellular context, rather than expression levels alone, determines NUSAP1's role in specific cancers. This tumor‐suppressive function of NUSAP1 in TNBC appears counterintuitive given its critical role in chromosome segregation and mitosis. Typically, ablation or dysfunction of such cell cycle regulators is incompatible with uncontrolled cancer cell proliferation. A similar paradox exists for cohesion, a protein complex that is essential for genome organization, chromosome segregation, and homologous recombination (HR)‐mediated DNA repair.^[^ ^44^, ^45^ ^]^ Although somatic mutations in cohesion subunits are recognized as human cancer drivers, they compromise chromosome segregation fidelity, leading to aneuploidy and whole‐chromosome instability.^[^ ^45^ ^]^ Notably, data from IntOgen (Integrative Onco Genomics) and COSMIC (Catalogue of Somatic Mutations in Cancer) identified over 27 mutation sites in the MAD of NUSAP1 across multiple tumors, including breast cancer (data not shown). This is particularly significant because MAD mediates the DAXX‐HIF interaction and NUSAP1 tumor‐suppressive function in TNBC. Key unanswered questions include whether elevated NUSAP1 expression in some cancers results from compensatory mechanisms induced by MAD mutations and which specific MAD mutations disrupt the DAXX‐HIF interaction in TNBC.

Since NUSAP1 itself is not a transcription factor, it is necessary to elucidate the mechanism of NUSAP1‐mediated HIF repression. Our data demonstrated that NUSAP1 bridges the transcriptional corepressor DAXX and HIF in the nucleus through its MAD, thereby suppressing HIF transcriptional activity in TNBC cells. NUSAP1 appears to have HIF‐independent tumor‐suppressive roles, which require further investigation. In contrast, NUSAP1 activates gene transcription in some cancer cell types. In pancreatic ductal adenocarcinoma (PDAC) cells, NUSAP1 promotes nuclear localization of HIF1α and c‐MYC, forming a transcription complex that enhances its transcriptional activity.^[^ ^46^ ^]^ Similarly, in lung cancer, lactate secreted by tumor cells induces NUSAP1 nuclear translocation in cancer‐associated fibroblasts (CAFs), enabling the JUNB‐FRA1‐FRA2 transcriptional complex to bind the DESMIN promoter and trigger IL‐8 secretion by CAFs, which recruit tumor‐associated macrophages (TAMs) and accelerate tumor growth.^[^ ^47^ ^]^ This inconsistency in NUSAP1's transcriptional effects may stem from tumor‐type specificity. Notably, the nuclear localization of NUSAP1 is crucial for its functions, as it contains a nuclear localization sequence (NLS) and directly binds to DNA.^[^ ^2^ ^]^ However, our data indicate that NUSAP1's native NLS (aa 186–211) alone cannot direct MAD to the nucleus (Figure S7B, Supporting Information). In contrast, a canonical SV40 NLS (PKKKRKV) enables efficient nuclear targeting of MAD without perturbing the microtubule structure. Intriguingly, NUSAP1's native NLS and MAD coordinately promoted microtubule bundling (Figure S7B, Supporting Information), although the underlying mechanism requires further elucidation. In this study, TS‐MAD (Flag‐tagged MAD with the canonical NLS) was designed to specifically repress HIF transcriptional activity in the nucleus, effectively inhibiting TNBC growth and metastasis. Given the prevalence of HIF1α and HIF2α overexpression in solid tumors, TS‐MAD may have broad applicability in cancers with elevated HIF1α and HIF2α levels. Thus, TS‐MAD represents a targeted, effective, and promising therapeutic strategy for HIF‐driven cancer. Due to the therapeutic potential of TS‐MAD, delivery approaches should be developed in the future. For example, synthesized TS‐MAD conjugated with a cell‐penetrating peptide can be directly administered via intratumoral or intravenous injection. Alternatively, the TS‐MAD peptide can be loaded into biodegradable polymeric nanoparticles for prolonged circulation and targeted release. Beyond these exogenous delivery methods, adeno‐associated virus (AAV) or oncolytic virus (OV) carrying the transgene for TS‐MAD can be utilized to produce the TS‐MAD internally.

Similar to NUSAP1, DAXX demonstrates a paradoxical pattern in cancer biology, while its expression is elevated in a variety of cancers and exhibits potent tumor‐suppressive effects.^[^ ^48^ ^]^ For instance, DAXX represses the expression of multiple pluripotent genes, including *SOX2*, *OCT4*, *NANOG*, *Notch4*, and *ALDH1A1*, thereby restricting cancer stem cell survival in ER+ breast cancer.^[^ ^49^ ^]^ Moreover, DAXX suppresses RAD51 transcription, impairs DNA damage repair, and sensitizes TNBC cells to DNA damage‐induced cell death.^[^ ^50^ ^]^ However, some studies have reported that DAXX promotes TNBC growth by enhancing *de novo* lipid synthesis,^[^ ^51^ ^]^ although the basis for these conflicting observations remains unclear. Although hypoxia represses DAXX expression in an HIF‐1‐dependent manner in lung cancer cells,^[^ ^17^ ^]^ its role in HIF regulation was previously unknown. Our data demonstrated that DAXX inhibits HIF transcriptional activity without altering HIF1α and HIF2α protein levels, establishing a double‐negative feedback loop between DAXX and HIF in TNBC progression. The precise mechanism underlying the DAXX‐mediated HIF regulation requires further investigation. Based on existing evidence, DAXX may indirectly modulate HIF transcriptional activity by facilitating heterochromatin assembly of HIF target genes. DAXX recruits histone methyltransferases (KMTs) to catalyze H3K9 methylation, thereby promoting heterochromatin assembly and transcriptional silencing.^[^ ^32^, ^33^, ^52^ ^]^ Although DAXX inhibits the DNA binding of several transcription factors through direct interaction with its DNA‐binding domain,^[^ ^53^, ^54^ ^]^ this mechanism does not apply to HIF regulation, as DAXX interacts with HIF1α’s ODD rather than its DNA‐binding domain.

## 4 Conclusion

Our findings demonstrate that NUSAP1 functions as a tumor suppressor in TNBC. Mechanistically, NUSAP1 bridges HIF and DAXX through its MAD, and DAXX subsequently recruits the methyltransferase SETDB1 to catalyze H3K9me3 deposition in the HRE regions of HIF target genes, thereby suppressing HIF transcriptional activity. Intriguingly, hypoxia impedes NUSAP1 transcription via HIF. Hence, NUSAP1 and HIF establish a double‐negative feedback loop that controls TNBC progression. Importantly, the TS‐MAD miniprotein was engineered to recapitulate NUSAP1's tumor‐suppressive functions, representing a promising therapeutic strategy for HIF‐driven TNBC.

## 5 Experimental Section

### Cell Culture

HEK293T (RRID: CVCL_0063), MDA‐MB‐231 (RRID: CVCL_0062), MDA‐MB‐468 (RRID: CVCL_0419), MCF10A (RRID:CVCL_0598), and HeLa (RRID: CVCL_0030) cells were obtained from the American Type Culture Collection (ATCC) with a certificate of analysis documenting the cell source, lot number, date of acquisition, and short tandem repeat (STR) profiling authentication. HCC1937 (CSTR:19375.09.3101HUMTCHu148) was purchased from the Cell Bank of the Chinese Academy of Sciences with a STR profiling report. All cell lines were cultured in DMEM (Macgene, CM15019) or RPMI 1640 (Macgene, CM10041) medium supplemented with 10% fetal bovine serum (FBS, ExCell, FSP500) or MCF10A cell complete medium (Procell, CM‐0525) at 37 °C with 5% CO_2_. Hypoxic cells were cultured in a modular incubator chamber (Stemcell Technologies), which was flushed with a gas mixture consisting of 1% O_2_, 5% CO_2_, and 94% N_2_. All cell lines were confirmed to be mycoplasma‐free as detected using the Mycolor One‐Step Mycoplasma Detector (Vazyme, D201‐01).

### Plasmids

Mammalian expression plasmids of the full‐length and truncated forms of human NUSAP1, HIF1α, DAXX, and SETDB1were obtained by PCR amplification and cloned into the lentiviral vectors pLVX‐IRES‐Flag, pLVX‐IRES‐HA, cFuGW‐Flag, or cFuGW‐Myc. Truncated NUSAP1, HIF1α, or DAXX cDNAs were subcloned into pGEX‐6P‐1 or pET‐28a vectors for protein purification. The NUSAP1 hypoxia‐response element (HRE) fragments were amplified from human genomic DNA and cloned into the pGL3‐Basic vector (Promega). The following primer pairs were used for HRE fragment amplification: HRE1 (Forward: 5′‐GATGGAGTTTCGCTCATTCCC‐3,’ Reverse: 5′‐CTGTCACTAAACTAGAGCACTTATCAACTC‐3′), HRE2 (Forward: 5′‐CGGAGTCTCGATCTGTCGTCC‐3,’ Reverse: 5′‐CGGAGTTAGACCCACAGCTCC‐3′), and HRE3 (Forward: 5′‐CATTTCATTAATACATCAAGTTCATATAAGAG‐3,’ Reverse: 5′‐GTGCGCGGACTCGGACTC‐3′). Wild‐type (WT) and mutant HRE‐3‐2 oligonucleotides were annealed and ligated into pGL3‐promoter vector (Promega) using the following primers: WT HRE3‐2 (Forward: 5′‐ GTACACACCGAGTCGGCGAGCACTGCGTGACACTGTGCGCACTGGAACACAGCGCACCG‐3′, Reverse: 5′‐ CTAGCGGTGCGCTGTGTTCCAGTGCGCACAGTGTCACGCAGTGCTCGCCGACTCGGTGT‐3′), mutant HRE3‐2 (Forward: 5′‐GTACACACCGAGTCGGCGAGCACTGCAAAACACTGTGCGCACTGGAACACAGCGCACCG‐3′, Reverse: 5′‐CTAGCGGTGCGCTGTGTTCCAGTGCGCACAGTGTTTTGCAGTGCTCGCCGACTCGGTGT‐3′) The shRNA sequences targeting human NUSAP1 (shRNA#1: 5′‐GAGCACCAAGAAGCTGAGAAT‐3′; shRNA#2: 5′‐CCTCAGGTAACAGAGATTCAA‐3′), DAXX (shRNA: 5′‐GAAGGGATGGACTAAGCTAAT‐3′), SETDB1 (shRNA: 5′‐ AGTTAGAGACATGGGTAATAC‐3′), and SUV39H1 (shRNA: 5′‐ AGTCGAGTACCTGTGCGATTA‐3′) were cloned into the pLKO.1 lentiviral vector. All the plasmids were confirmed by DNA sequencing. HIF1α knockout (HIF1α‐KO), HIF2α‐KO, and HIF1α/HIF2α double knockout (HIF‐DKO) plasmids have been previously described.^[^ ^25^, ^34^ ^]^ Other plasmids, including pLV3‐CMV‐KHDRBS1(human)‐EGFP‐Puro (P54110), pCMV‐EGFP‐SETDB1‐Neo (P68558), and pLV3‐CMV‐SUV39H1‐3 × FLAG‐Puro (P47864), were purchased from MiaoLing Plasmid Platform (https://en.miaolingbio.com/).

### Lentivirus Production and Establishment of Stable Cell Lines

The lentivirus was generated as previously described.^[^ ^55^ ^]^ Briefly, HEK293T cells were co‐transfected with individual expression plasmids and packaging vectors pMD2.G and psPAX2 using Polyjet transfection reagent (SignaGen, SL100688). Forty‐8 h after transfection, the virus particles were collected, filtered using a 0.45 µm syringe filter, and then transduced into breast cancer cells for co‐culture for 48 h. Subsequently, selection was performed using puromycin dihydrochloride (1.0 µg mL^−1^, Abcam, ab141453) or G‐418 disulfate (3.0 mg mL^−1^, MedChemExpress, HY‐17561) for 4–8 days. HIF1α‐KO, HIF2α‐KO, HIF‐DKO, and other breast cancer cell lines have been previously described.^[^ ^25^, ^34^ ^]^ Notably, the shNUSAP1, shDAXX, and TS‐MAD cell lines were polyclonal, whereas the HIF1α‐KO, HIF2α‐KO, and HIF‐DKO cell lines were single‐cell clones. NUSAP1 was depleted in HIF‐DKO (clone #30) MDA‐MB‐231 and HIF‐DKO (clone #5) MDA‐MB‐468 cells to generate shNUSAP1 + HIF‐DKO and shDAXX + HIF‐DKO cell lines.

### Antibodies and Reagents

MG132 (ab141003), anti‐H3K9me3 (ab8898; RRID: AB_306848) were obtained from Abcam. Normal mouse IgG (sc‐2025; RRID: AB_737182) and anti‐DAXX (sc‐8043 (H‐7); RRID: AB_627405) antibodies were obtained from Santa Cruz Biotechnology. Anti‐Flag‐M2 antibody (F1804; RRID: AB_262044) was obtained from Sigma‐Aldrich. Anti‐NUSAP1 antibody (12024‐1‐AP; RRID: AB_2 157 789), anti‐HA antibody (51064‐2‐AP; RRID: AB_11 042 321), anti‐β‐actin antibody (66009‐1‐Ig; RRID: AB_2 687 938), anti‐Myc antibody (16286‐1‐AP; RRID: AB_11 182 162), anti‐GFP antibody (66002‐1‐Ig; RRID: AB_11 182 611), anti‐GAPDH antibody (10494‐1‐AP; RRID: AB_2 263 076), anti‐Ki‐67 antibody (27309‐1‐AP, RRID: AB_2 756 525), HRP‐conjugated affinipure goat anti‐mouse secondary antibody (SA00001‐1; RRID: AB_2 722 565), anti‐SETDB1 (11231‐1‐AP; RRID:AB_2 186 069), anti‐SUV39H1 (10574‐1‐AP; RRID:AB_2 196 710), and horseradish peroxidase (HRP)‐conjugated affinipure goat anti‐rabbit secondary antibody (SA00001‐2; RRID: AB_2 722 564) were obtained from Proteintech. Anti‐HIF1α (A300‐286A; RRID: AB_2 117 114) and anti‐HIF2α antibodies (A700‐003; RRID: AB_2 631 884) were obtained from Bethyl. Anti‐HIF1β antibody (NB100‐124; RRID: AB_10 077 794) was purchased from NOVUS. Normal rabbit IgG antibody (2729; RRID: AB_1 031 062) and anti‐cleaved caspase 3 (Asp175) antibody (9661; RRID: AB_2 341 188) were obtained from Cell Signaling Technology. Alexa Fluor 488‐conjugated donkey anti‐rabbit secondary antibody (A21206; RRID: AB_2 535 792) and Alexa Fluor 594‐conjugated donkey anti‐mouse secondary antibody (A21203; RRID: AB_141 633) were obtained from Thermo Fisher Scientific.

### Immunofluorescence Staining

Immunofluorescence staining was performed as previously described.^[^ ^56^, ^57^ ^]^ Briefly, breast cancer cells or HeLa cells were washed twice with PBS buffer and then fixed with 4% paraformaldehyde (PFA, Sangon Biotech, E672002) for 10 min at room temperature. The cells were then washed thrice with PBS. Permeabilization was performed using PBS buffer containing 0.2% Triton X‐100 (Sangon Biotech, A600198) for 15 min, followed by blocking with 4% BSA (Sangon Biotech, A602440) for 30–60 min. Subsequently, the cells were incubated overnight at 4 °C with anti‐HIF1α, anti‐HIF2α, anti‐NUSAP1, anti‐FLAG, and anti‐α‐tubulin antibodies prepared in 2% BSA. The next day, the cells were washed three times with PBS and then incubated with secondary fluorescently labeled antibodies for 1 h at room temperature. Thereafter, cells were stained with DAPI (Sigma‐Aldrich, 10 236 276 001) for nuclear staining and mounted on slides. Immunofluorescence images were acquired using a TCS SPE confocal microscope (Leica).

### Cell Proliferation Assay

A total of 1 × 10⁴ breast cancer cells were seeded into 24‐well plates (Jet Bio‐Filtration, TCP011024) in triplicates. The cells were cultured in hypoxic and normoxic incubators for 24, 48, or 72 h. The number of cells at each time point was counted to assess the cell proliferation rate.

### Colony Formation Assay

One hundred breast cancer cells were seeded into 12‐well plates (Jet Bio‐Filtration, TCP011012) in triplicate and cultured under normoxic (20% O_2_) or hypoxic (1% O_2_) conditions for 14 days until distinct cell colonies were visible. The colonies were gently washed twice with PBS and fixed with 4% paraformaldehyde (PFA) for 10 min. After washing twice with PBS, colonies were stained with 0.5% crystal violet (Beyotime, C0121) for 10 min. Finally, the colonies were continuously washed with PBS until no more color was removed from the colonies, and the number of colonies was counted.

### Boyden Chamber Assay

A total of 5 × 10^4^ breast cancer cells were resuspended in a serum‐free medium. The cells were then seeded into the upper chambers (Jet Bio‐Filtration, TCS003024) (for the migration assay) or Matrigel (Corning, 354 234)‐coated upper chambers (for the invasion assay). Subsequently, they were cultured under normoxic (20% O_2_) or hypoxic (1% O_2_) conditions for 16 or 24 h for migration and invasion assays, respectively. Cells that migrated or invaded the lower surface of the Transwell insert were fixed with methanol, stained with 0.5% crystal violet, and counted.

### Immunoprecipitation and Western Blotting

Cells were resuspended and sonicated in NETN lysis buffer (10 mM Tris‐HCl, 150 mM NaCl, 1 mM EDTA, 0.5% NP‐40, pH 7.4, and protease inhibitor cocktail), followed by centrifugation. The supernatant was subjected to immunoblotting (IB) analysis. For immunoprecipitation assays, the supernatant was incubated with the indicated antibodies and protein A/G magnetic beads (B23202; Selleck) overnight at 4 °C. Subsequently, the beads were gently washed three times with NETN lysis buffer, and the immunoprecipitates were resuspended in 1 × Laemmli buffer and boiled for 6 min. Protein–protein interactions were detected by SDS‐PAGE.

### Liquid Chromatography‐Tandem Mass Spectrometry (LC‐MS/MS) Assay

MDA‐MB‐231 cells stably expressing Flag‐NUSAP1, treated with 10 µm MG132 for 6 h before harvest, were lysed using NETN lysis buffer. The supernatant was collected and incubated with anti‐Flag magnetic beads (BioLinkedIn, L1103) at 4 °C for 2 h. The putative NUSAP1‐interacting proteins in the immunoprecipitates were fractionated by SDS‐PAGE and subjected to LC‐MS/MS analysis conducted by PTM Biolabs Inc. Briefly, the tryptic peptides were analyzed using an NSI source, followed by MS/MS in a Q Exactive Plus (Thermo) system coupled online to the EASY‐Nlc1000 UPLC system. The resulting MS/MS data were processed using Proteome Discoverer 1.3. Tandem mass spectra were searched against the Homo Sapiens database (SwissProt, 20 395 sequences). Trypsin/P was specified as the cleavage enzyme, allowing up to 2 missing cleavages. Mass error tolerance was set to 10 ppm for precursor ions and 0.02 Da for fragment ions. Carbamidomethylation on cysteine residues was specified as a fixed modification, and oxidation on methionine residues was specified as a variable modification. Peptide confidence was set to high, and the peptide ion score threshold was set to > 20.

### Protein Purification and Pull‐Down Assay

The GST‐ and His‐fusion proteins were purified as previously described.^[^ ^57^ ^,58]^ Briefly, GST‐HIF1α‐ODD, 6 × His‐DAXX‐HBD, or 6 × His‐NUSAP1‐MAD were expressed in BL21 competent *E. coli* cells and purified with glutathione‐Sepharose 4 B (Cytiva, 17‐0756‐01) or HisSep Ni‐NTA MagBeads (Yeasen, 20561ES08) according to standard protocols. Purified GST or GST‐fusion proteins were incubated with purified His‐fusion proteins and glutathione‐Sepharose 4B beads. Subsequently, the beads were washed extensively five times before boiling, and western blotting was performed to analyze the proteins bound to the beads.

### RT‐qPCR

Total RNA was extracted from breast cancer cells using the TRIzol reagent (Invitrogen, 11 596 018) and treated with DNase I (Ambion, AM2224), followed by reverse transcription using a reverse transcription kit (Yeasen, 11141ES10). RT‐qPCR assays were performed using a CFX‐96 real‐time system (Bio‐Rad) with SYBR Green Master Mix (Yeasen, 11201ES08), according to the standard protocol.^[^ ^39^ ^]^ The following primer pairs were used for RT‐qPCR: *CCND2* (Forward: 5′‐TTTGCCATGTACCCACCGTC‐3′, Reverse: 5′‐AGGGCATCACAAGTGAGCG‐3′), *PDE2A* (Forward: 5′‐CCTGCGCCTTCAACAAGCTA‐3′, Reverse: 5′‐GGCCTCCGTGATGATCTCCT‐3′), *NDNF* (Forward: 5′‐ATCAGCCATACCCTGAGTTACC‐3′, Reverse: 5′‐TGAATGGGTTGTTTCAGCAAAGA‐3′), *LOXL4* (Forward: 5′‐TCGTGGCTACCTTTCTGAAAC‐3′, Reverse: 5′‐GTGGCCCTCATACTTCACCTC‐3′), *ANGPTL4* (Forward: 5′‐GTCCACCGACCTCCCGTTA‐3′, Reverse: 5′‐CCTCATGGTCTAGGTGCTTGT‐3′), *BBC3* (Forward: 5′‐GACCTCAACGCACAGTACGAG‐3′, Reverse: 5′‐AGGAGTCCCATGATGAGATTGT‐3′), *PMAIP1* (Forward: 5′‐CATGAGGGGACTCCTTCAAA‐3′, Reverse: 5′‐TTCCATCTTCCGTTTCCAAG‐3′), *PPARD* (Forward: 5′‐CAGGGCTGACTGCAAACGA‐3′, Reverse: 5′‐CTGCCACAATGTCTCGATGTC‐3′), *Vimentin* (Forward: 5′‐GACGCCATCAACACCGAGTT‐3′, Reverse: 5′‐CTTTGTCGTTGGTTAGCTGGT‐3′), and 18s rRNA (Forward: 5′‐CGGCGACGACCCATTCGAAC‐3′, Reverse: 5′‐ GAATCGAACCCTGATTCCCCGTC‐3′). The fold‐change in mRNA expression was calculated based on the threshold cycle (Ct) using the formula 2^−Δ(ΔCt)^, where ΔCt = Ct_target_ – Ct_18S RNA_ and Δ(ΔCt) = Ct_1% O2_ – Ct_20% O2_.

### Luciferase Reporter Assay

Luciferase reporter assays were conducted as previously described.^[^ ^34^ ^]^ Briefly, breast cancer cells were seeded in 48‐well plates and transfected with HIF luciferase reporter plasmid (p2.1, which contains 2 × HREs from human *ENO1*, a shared target gene of HIF1 and HIF2) or *NUSAP1* HRE luciferase reporter plasmids, control reporter plasmid pSV‐Renilla, and shRNA or overexpression plasmids using Lipofectamine 3000 (Invitrogen, L3000015). 24 h after transfection, cells were cultured under normoxic or hypoxic conditions for 24 or 48 h. The luciferase activities of firefly and Renilla were detected using the Dual‐Luciferase Reporter Assay System kit (Promega, E1910) and Centro LB 960 microplate luminometer (Berthold, Centro LB 960).

### Chromatin Immunoprecipitation (ChIP)‐qPCR

ChIP assays were performed using the Hyperactive pG‐MNase CUT & RUN Assay Kit according to the manufacturer's instructions. Briefly, 1 × 10⁷ breast cancer cells were collected and resuspended in wash buffer, followed by incubation with Concanavalin A‐coated Magnetic Beads Pro (CoA Beads Pro) for 10 min at room temperature. Low‐speed centrifugation was conducted for rapid spin‐down of the cell‐CoA Beads Pro complex, which was then incubated with the primary antibody and MNase‐conjugated protein G (pG‐MNase) at 4 °C for 2 and 1 h, respectively. The cells were permeated by adding digitonin to the reaction system to facilitate MNase‐mediated DNA fragmentation. DNA fragments were collected using FastPure DNA Mini columns and analyzed by qPCR assays using the following primers: *NUSAP1* (forward, 5′‐CTCTCGGGCTAGCCTC‐3′, reverse: 5′‐GTGGGATACGCAGGTGGTG‐3′), *ANGPTL4* (forward, 5′‐CTCTCGGGCTAGCCTC‐3′, reverse: 5′‐GTGGGATACGCAGGTGGTG‐3′), *FASN* (Forward, 5′‐TAGAGGGAGCCAGAGAGACG‐3′, reverse: 5′‐GCTGCTCGTACCTGGTGAG‐3′), and p21 (Forward, 5′‐GTGGCTCTGATTGGCTTTCTG‐3′, reverse: 5′‐CTGAAAACAGGCAGCCCAAG‐3′).

### RNA Sequencing (RNA‐Seq)

Parental, HIF‐DKO, and shNUSAP1#1 MDA‐MB‐231 cells were cultured under 20% O_2_ or 1% O_2_ conditions for 24 h. Total RNA was isolated using the RNeasy Mini Kit and treated with DNase (Qiagen), followed by quantification using an Agilent 2100 Bioanalyzer (Agilent). Poly(A)^+^ RNA was enriched using VAHTS mRNA Capture Beads 2.0 (Vazyme, N403‐02) and then subjected to library preparation using the Optimal Dual‐mode mRNA Library Prep Kit (Beijing Genomics Institute, BGI). Briefly, mRNA with poly (A) was purified and fragmented to generate first‐strand cDNA, followed by second‐strand synthesis. The cDNA was sequentially subjected to end repair, A‐tailing addition, adaptor ligation, purification, and amplification. Finally, the libraries were sequenced using an Illumina sequencing platform with a read configuration of 50 bp and a single end. Raw sequencing data were filtered using SOAPnuke to generate clean data. The clean data were aligned using the spliced read aligner HISAT2, which was supplied with the GCF_0 00001405.39_GRCh38.p13 human genome assembly as the reference genome. Gene expression levels were calculated by RSEM (RNA‐Seq by Expectation Maximization (RSEM). Genes were annotated using the GENCODE database. Differential gene expression results were analyzed under the conditions of false discovery rate (FDR) < 0.05, and mRNA fold change ≥ 2. The differentially expressed genes were functionally classified, and the phyper in R software was used for KEGG enrichment analysis, with a *p* value < 0.05.

### Animal Studies

Female NOD‐SCID mice aged 6–8 weeks (GemPharmatech, T001492) were used for the xenograft studies. 2 × 10⁶ MDA‐MB‐231 or MDA‐MB‐468 cells were resuspended in a 1:1 mixture of 50 µL 1 × PBS and 50 µL Matrigel, and then injected into the second left mammary fat pad of the mice. Once the tumors grew to a measurable size, the tumor volume was measured every three days using a caliper and analyzed according to the formula: V = 0.52 × L × H × W (where V is the volume, L is the length, H is the height, and W is the width). When the volume of the primary tumors reached ≈1500 mm^3^, the mice were euthanized with carbon dioxide. The lungs were perfused with 1 × PBS, inflated with 0.1% agarose (Sangon Biotech, A600015), and fixed with formalin (Sangon Biotech, E672001). Tumors were surgically removed, weighed, and photographed. For tail vein injection, 1 × 10^6^ cells resuspended in 100 µL of 1 × PBS were slowly injected into female NOD‐SCID mice via the tail vein. Three weeks later, the lungs were perfused with 1 × PBS and harvested. All mice were housed in a specific pathogen‐free (SPF) facility with the approval of the Animal Care and Use Committee of Shandong Normal University (ethics approval number AEECSDNU2023014).

### H&E Staining

Tissue samples were fixed in formalin for 24 h, followed by paraffin embedding and sectioning. The sections on the slides were dewaxed with xylene (Biosharp, BL170A) and then hydrated with gradient ethanol. Subsequently, staining was performed using a hematoxylin and eosin staining kit (Solarbio, G1120). Specifically, the sections were stained with hematoxylin for 5–10 min, washed with water, differentiated with 1% hydrochloric acid ethanol, and stained with eosin for 2–5 min. After staining, the sections were dehydrated with gradient ethanol, cleared with xylene, and mounted with neutral gum (Solarbio, G8590). After completing the above steps, the sections were observed under a microscope equipped with a digital camera, and images were taken for analysis.

### Immunohistochemistry (IHC)

Tissue samples were fixed in formalin solution for 24 h, followed by paraffin embedding and sectioning. The sections were then placed on the slides. First, they were dewaxed with xylene (Biosharp, BL170A) and hydrated with gradient ethanol. Subsequently, antigens were retrieved from the solution using a citrate antigen retrieval solution (Beyotime Biotech, P0083) by heating for 10 min. After cooling to room temperature, samples were treated with 3% hydrogen peroxide for 10 min to block endogenous peroxidase activity. The samples were then rinsed with PBS buffer and blocked with a 5% BSA blocking solution containing 0.1% Triton X‐100 for 1 h. After blocking, the samples were incubated overnight with primary antibodies prepared in 3% BSA. The following primary antibodies were used: anti‐Ki‐67 (1:5000 dilution) and anti‐cleaved caspase‐3 (CC3; 1:500 dilution). The following day, the samples were incubated with horseradish peroxidase‐conjugated secondary antibodies for 1.5 h. Color development was performed using DAB chromogenic solution (Solarbio, DA1010), and the cell nuclei were stained with hematoxylin. After staining, samples were dehydrated with gradient ethanol, cleared with xylene, and mounted with neutral gum (Solarbio, G8590). After completing the above operations, the samples were observed under a microscope equipped with a digital camera, and photos were taken for analysis.

### Statistical Analysis

All experiments were performed with three independent biological replicates unless otherwise stated. Statistical analyses were performed using one‐way or two‐way ANOVA with multiple testing correction for comparisons among multiple groups, a two‐tailed *t*‐test for comparisons between two groups, or a two‐tailed Pearson correlation test (GraphPad Prism 8.0). Data were presented as the mean ± standard deviation (SD). Statistical significance was defined as ^*^ *P* < 0.05, ^**^ *P* < 0.01, ^***^ *P* < 0.001, and ^****^ *P* < 0.0001.

## Conflict of Interest

The authors declare no conflict of interest.

## Author Contributions

Y.T.D., J.J.W., M.W., and Y.Z. contributed equally to this work. Y.J.W., J.Z., and Y.C. conceived the study, analyzed the data, and wrote and edited the manuscript. Y.T.D. performed most experiments, analyzed the data, and wrote the manuscript. J.J.W. conducted mouse breeding, fat pad injections, tumor measurements and isolation, IHC staining, and wrote the manuscript. M.W. performed the luciferase reporter assays, RNA‐seq, RT‐qPCR, and ChIP‐qPCR. Y.Z. generated cell lines, performed cell proliferation, migration, and invasion assays, and wrote the manuscript. M.M.Z., H.Y.L, X.M.W, and K.X.T generated plasmids, purified His‐ and GST‐fusion proteins, and performed immunostaining assays. H.R.S, C.L.S., and Q.Z. performed bioinformatics analysis. X.Q.M. provided key technical mentoring.

## Supporting information



Supporting Information

## Data Availability

The GSE108833 and GSE18494 datasets, which document hypoxia‐responsive gene expression profiles in breast cancer cells, were downloaded from the Gene Expression Omnibus database (GEO, https://www.ncbi.nlm.nih.gov/geo/). mRNA expression levels of *NUSAP1*, *CCND2*, *PDE2A*, *NDNF*, *LOXL4*, and *ANGPTL4* in human breast tumors and normal breast tissues were acquired from the Biomarker Exploration for Solid Tumors (BEST, https://rookieutopia.com/) database and R2 Genomics Analysis and Visualization Platform (http://r2.amc.nl), respectively. Kaplan–Meier survival analysis for patients with breast cancer was performed using datasets downloaded from the PrognoScan database (http://www. prognoscan. org). The raw RNA sequencing (RNA‐seq) data generated in this study were deposited in the National Center for Biotechnology Information (NCBI) database under accession number PRJNA1244068. The mass spectrometry raw data have been deposited to the ProteomeXchange Consortium via the PRIDE partner with the dataset identifier PXD065054. All data generated or analyzed during this study are included in this article and its supplementary information files. Additional data presented in this paper are available from the corresponding author upon reasonable request.
