# Supplementary material for: NUSAP1 Recruits DAXX to Suppress HIF‐Driven Triple‐Negative Breast Cancer Progression
Source: Adv Sci (Weinh). 2025 Nov 3;13(5):e13380. doi: 10.1002/advs.202513380 (PMC12850156; doi:10.1002/advs.202513380)
Supplement: Supplementary file 1 — Supporting Information [file ADVS-13-e13380-s001.docx]

**Supporting Information**

**NUSAP1 Recruits DAXX to Suppress HIF-driven Triple-negative Breast Cancer Progression**

*Yating Du, Jingjing Wang, Min Wang, Yao Zhang, Miaomiao Zheng, Huiyan Li, Xuemeng Wang, Huanran Sun, Kexin Tang, Changliang Shan, Qiang Zhao, Xiaoqian Meng, Yijie Wang^*^, Jun Zhou^*^, Yan Chen^*^*


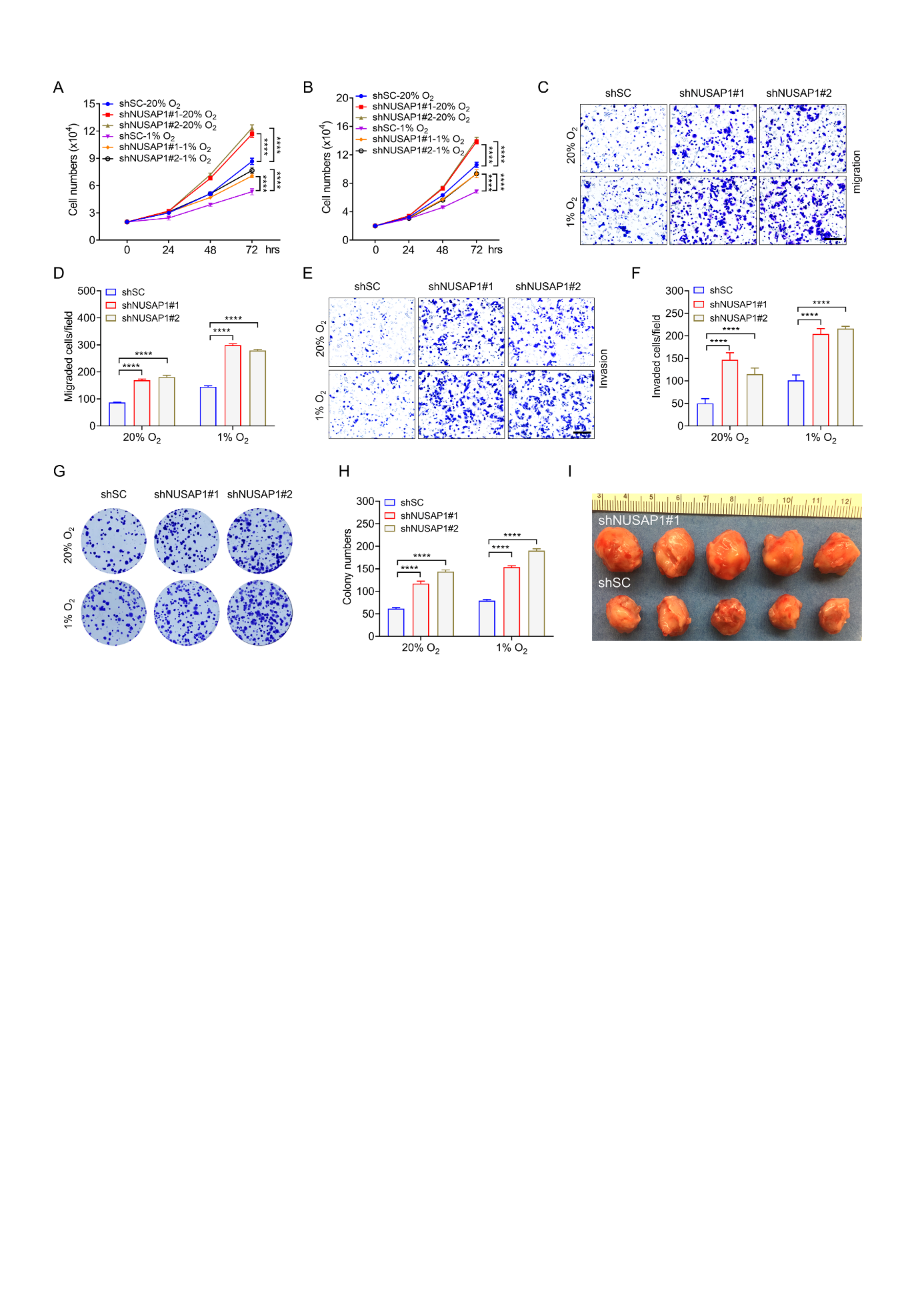


**Figure S1.** NUSAP1 functions as a tumor suppressor in TNBC. A,B) Cell growth rate of the indicated MDA-MB-231 (A) and MDA-MB-468 (B) breast cancer cell lines exposed to 20% or 1% O_2_ for 0, 24, 48, and 72 hours. C-F) The capacity of shSC and shNUSAP1 MDA-MB-468 cells in migration (C,D) and invasion (E,F) was examined by Boyden chamber assays. Scale bar, 60 μm. G,H) The survival of shSC and shNUSAP1 MDA-MB-468 cells was determined by colony formation assays. I) Image of the indicated xenograft breast tumors. n = 5 mice per group. *****P* < 0.0001, by 2-way ANOVA Tukey’s multiple comparisons test (A,B,D,F,H).


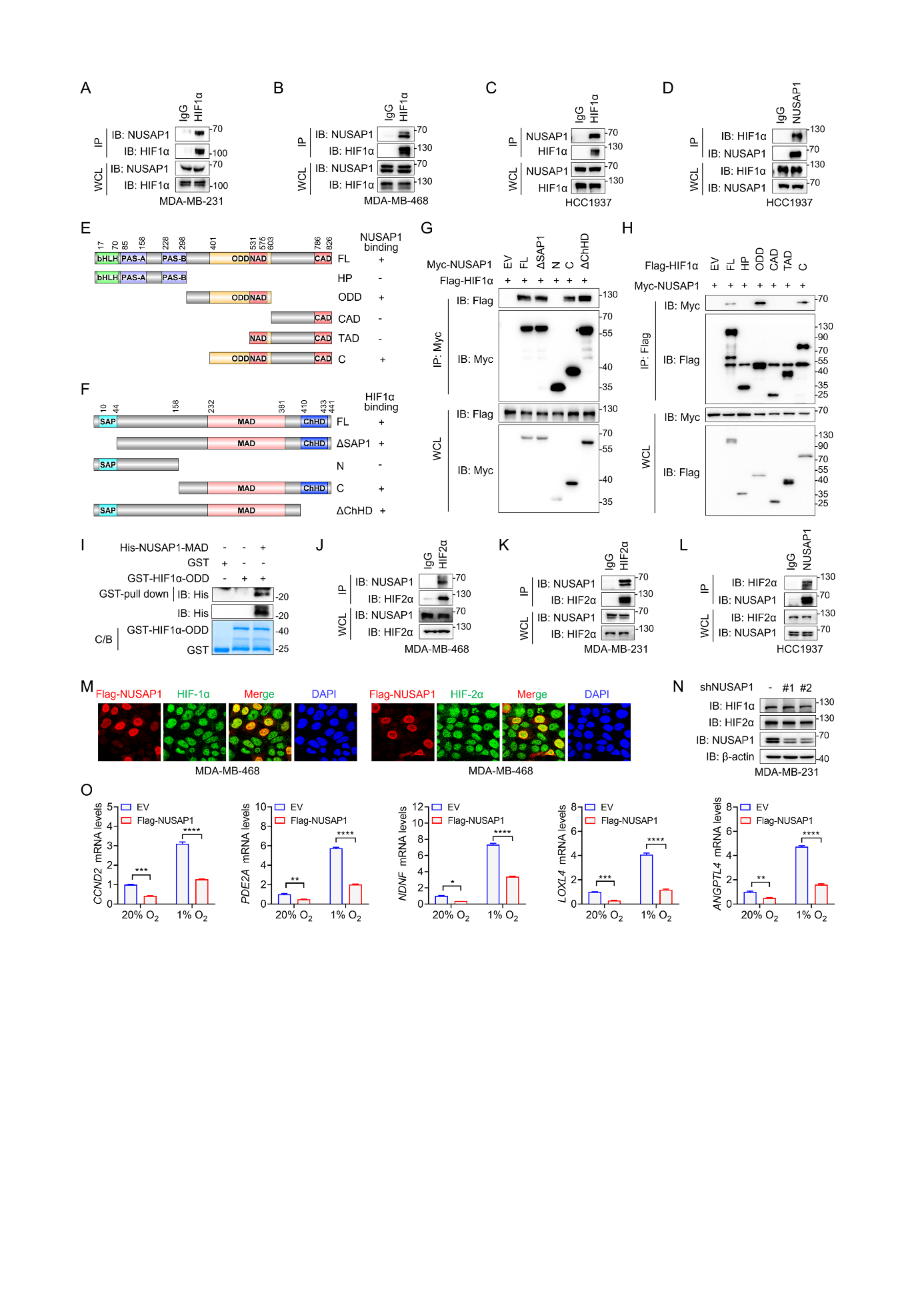


**Figure S2.** NUSAP1 associates with HIFα and restricts its transcriptional activity. A-D) The interaction between HIF1α and NUSAP1 in MDA-MB-231, MDA-MB-468, and HCC1937 cells treated with MG132 for 6 hours before harvest. E-H) Mapping of the domains in NUSAP1 and HIF1α that mediate the NUSAP1-HIF1α interaction. I) Pull down assays using purified MAD of NUSAP1 and ODD of HIF1α. C/B, Coomassie blue staining. J-L) The interaction between HIF2α and NUSAP1 in MDA-MB-468 (J), MDA-MB-231 (K), and HCC1937 (L) TNBC cells. M) Immunostaining of ectopically expressed Flag-NUSAP1 and endogenous HIF1α or HIF2α in MDA-MB-468 cells exposed to hypoxia for 6 hours. N) The protein levels of HIF1α and HIF2α in NUSAP1-depleted MDA-MB-231 cells. O) The mRNA levels of HIF target genes were determined by RT-qPCR in indicated MDA-MB-468 cells exposed to 20% or 1% O_2_ for 24 hours. **P* < 0.05, ***P* < 0.01, ****P* < 0.001, *****P* < 0.0001, by 2-way ANOVA Tukey’s multiple comparisons test (O).


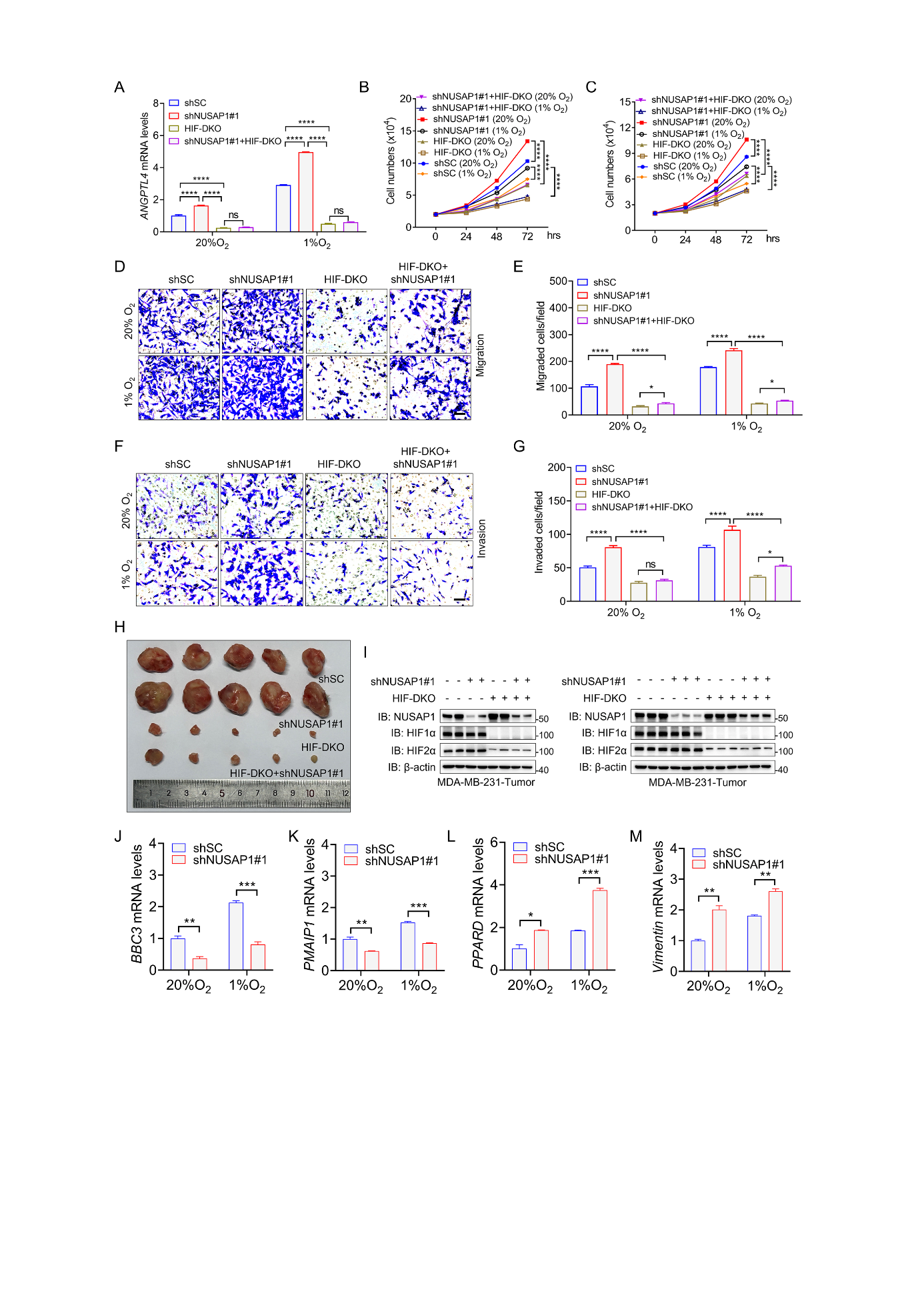


**Figure S3.** HIF-mediated oncogenic potential of TNBC cells is attenuated by NUSAP1. A) RT-qPCR assays in the indicated MDA-MB-231 breast cancer cell lines exposed to 20% or 1% O2 for 24 hours. B,C) The growth rates of indicated MDA-MB-231 (B) and MDA-MB-468 (C) cell lines exposed to 20% or 1% O_2_ for 24, 48, and 72 hours. D-G) The abilities of MDA-MB-468 cell lines in migration (D,E) and invasion (F,G). Scale bar, 60 μm. H) Image of the indicated xenograft breast tumors. n = 5 mice per group. I) The protein levels of HIF1α, HIF2α, and NUSAP1 in indicated xenograft breast tumors. J-M) The mRNA levels of p53 (J,K) and Wnt (L,M) target genes were determined in shSC and shNUSAP1#1 MDA-MB-231 cells exposed to 20% or 1% O_2_ for 24 hours. **P* < 0.05, ***P* < 0.01, ****P* < 0.001, *****P* < 0.0001, by 2-way ANOVA Tukey’s multiple comparisons test (A-C,E,G,J-M). ns, no significance.


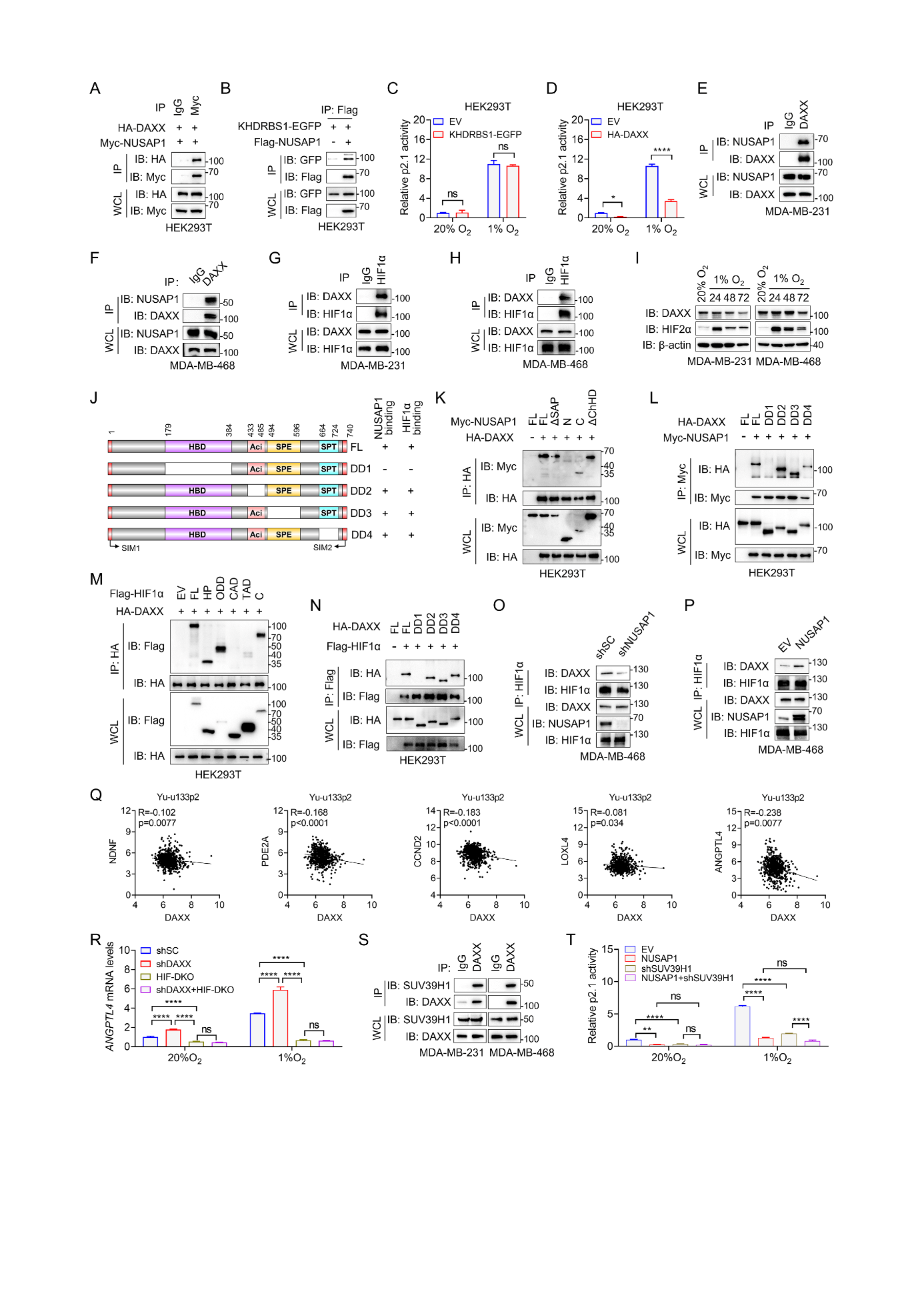


**Figure S4.** NUSAP1 suppresses HIF by recruiting transcriptional corepressor DAXX. A) Interaction between Myc-NUSAP1 and HA-DAXX in HEK293T cells. B) Interaction between Flag-NUSAP1 and KHDRBS1-EGFP in HEK293T cells. C,D) HEK293T cells transfected with EV (empty vector), KHDRBS1-EGFP (C), or HA-DAXX (D) together with reporter plasmids were exposed to 20% or 1% O_2_ for 24 hours and then subjected to luciferase reporter assays. E-H) The interaction between endogenous DAXX and NUSAP1 (E,F) or DAXX and HIF1α (G,H) in MDA-MB-231 or MDA-MB-468 cells. I) DAXX protein levels were analyzed in MDA-MB-231 and MDA-MB-468 cells exposed to either 20% O_2_ or 1% O_2_ for 6 hours. J-N) Mapping of domains that mediate the interaction of DAXX with NUSAP1 and HIF1α in HEK293T cells. O,P) The DAXX-HIF1α interaction was detected in MDA-MB-468 cells with NUSAP1 ablation (O) or overexpression (P). Q) The correlation of mRNA levels of DAXX and indicated HIF target gens in human breast tumors and normal breast tissues. R) RT-qPCR assays in the indicated MDA-MB-231 breast cancer cell lines exposed to 20% or 1% O2 for 24 hours. S) Interaction between SUV39H1 and DAXX in MDA-MB-231 and MDA-MB-468 cells. T) Luciferase reporter assays in the indicated MDA-MB-231 cell lines exposed to 20% O_2_ or 1% O_2_ for 24 hours. **P* < 0.05, ***P* < 0.01, *****P* < 0.0001, by 2-way ANOVA Tukey’s multiple comparisons test (C,D,R,T). ns, no significance.


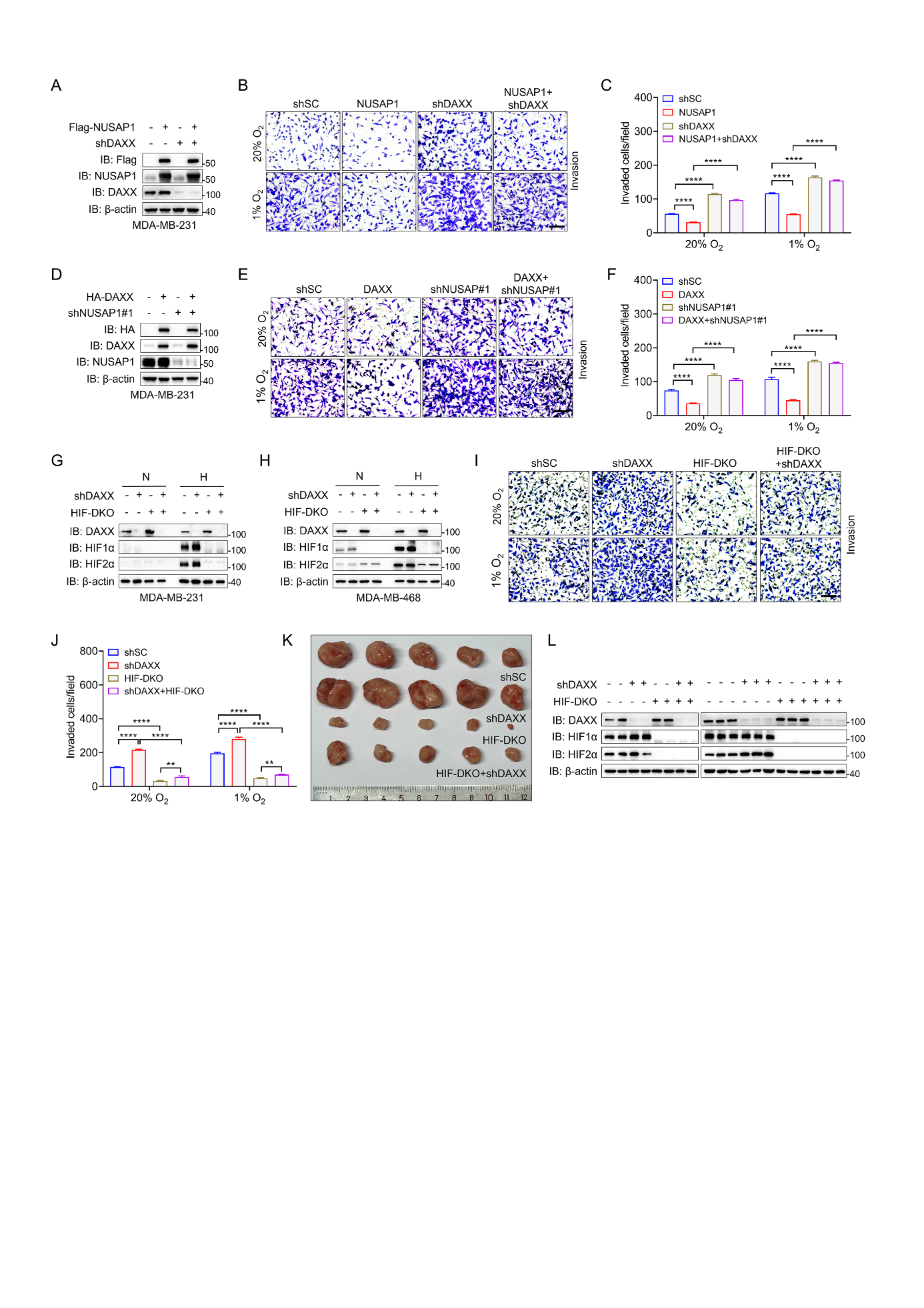


**Figure S5.** DAXX and NUSAP1 coordinately suppress HIF-mediated TNBC progression. A) The protein levels of NUSAP1 and DAXX in MDA-MB-231 cells. B,C) The ability of indicated MDA-MB-231 cells in invasion. Scale bar, 60 μm. D) The protein levels of NUSAP1 and DAXX in MDA-MB-231 cells. E,F) The capacity of indicated MDA-MB-231 cells in invasion. Scale bar, 60 μm. G,H) DAXX was knocked down in HIF-DKO MDA-MB-231 (clone #30) and HIF-DKO MDA-MB-468 (clone #5) cell lines, respectively. Protein levels of HIF1α, HIF2α, and DAXX were examined in indicated MDA-MB-231(G) and MDA-MB-468 (H) cell lines exposed to 20% or 1% O_2_ for 6 hours. I,J) The capacity of indicated MDA-MB-468 cells in invasion. Scale bar, 60 μm. K) Image of the indicated xenograft breast tumors. n = 5 mice per group. L) The protein levels of HIF1α, HIF2α, and DAXX in indicated xenograft breast tumors. ***P* < 0.01, *****P* < 0.0001, by 2-way ANOVA Tukey’s multiple comparisons test (C,F,J).


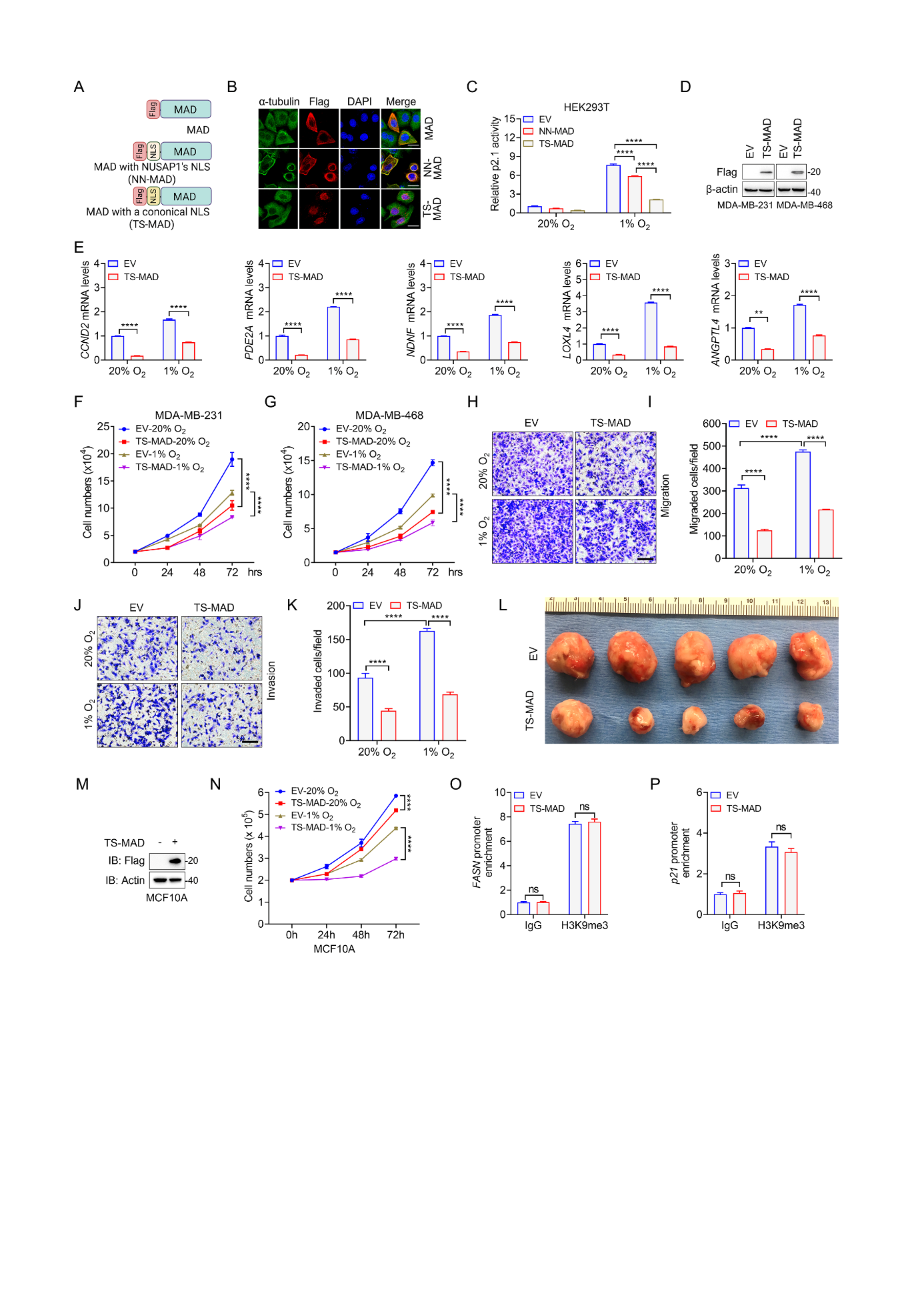


**Figure S6.** TS-MAD suppresses TNBC progression through HIF inhibition. A) Schematic depiction of engineered MAD, NN-MAD, and TS-MAD. NLS, nuclear localization sequence; NN, NUSAP1’s NLS; TS, tumor suppressor; MAD, microtubule-associated domain. B) Immunostaining of microtube and MAD, NN-MAD, or TS-MAD in HeLa cells. C) The effect of ectopically expressed NN-MAD and TS-MAD on HIF luciferase reporter activity in HEK293T cells exposed to 20% or 1% O_2_ for 24 hours. D) The protein levels of TS-MAD in MDA-MB-231 and MDA-MB-468 cells. E) The mRNA levels of indicated HIF target genes were determined by RT-qPCR in EV and TS-MAD MDA-MB-468 cells exposed to 20% or 1% O_2_ for 24 hours. F,G) Growth rate of indicated MDA-MB-231 and MDA-MB-468 cell lines exposed to 20% or 1% O_2_ for 0, 24, 48, and 72 hours. H-K) The abilities of indicated MDA-MB-468 cells in migration (H,I) and invasion (J,K). Scale bar, 60 μm. L) Image of the indicated xenograft breast tumors. n = 5 mice per group. M,N) Growth rate of indicated MCF10A cell lines exposed to 20% or 1% O_2_ for 0, 24, 48, and 72 hours. O,P) ChIP assays were performed in normoxic MDA-MB-231 cells using indicated antibodies, followed by qPCR analysis. ***P* < 0.01, *****P* < 0.0001, by 2-way ANOVA Tukey’s multiple comparisons test (C,E-G,I,K,N-P).


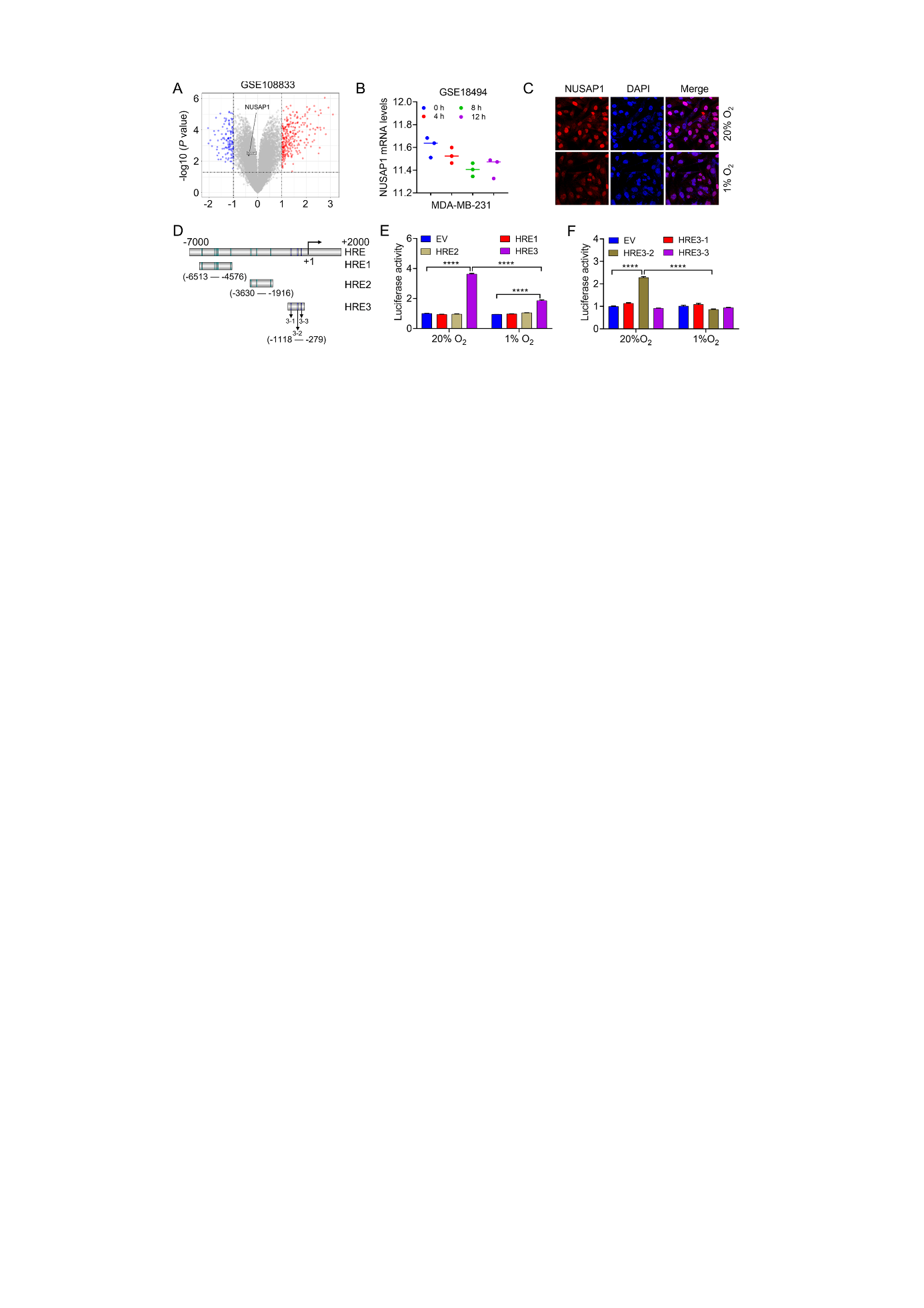


**Figure S7.** NUSAP1’s expression is repressed by HIF in breast cancer cells. A) GEO data analysis (GSE108833) displaying differentially expressed genes (volcano plot) in MDA-MB-231 cells exposed to 20% or 1% O_2_ for 24 hours. B) GEO data analysis (GSE18494) showing NUSAP1’s mRNA levels in MDA-MB-231 cells exposed to 1% O_2_ for 0, 4, 8, or 12 hours. C) Immunostaining of NUSAP1 in MDA-MB-231 cells exposed to 20% or 1% O_2_ for 48 hours. D) Schematic representation of putative hypoxia response elements (HREs) in *NUSAP1*’s promoter (7 kb). HRE1, -6513 bp ─ -4576 bp; HRE2, -3630 bp ─ -1916 bp; HRE3, -1118 bp ─ -279 bp. E,F) Luciferase reporter assays in HEK293T cells transfected with indicated reporter plasmids and exposed to 20% or 1% O_2_ for 24 hours. *****P* < 0.0001, by 2-way ANOVA Tukey’s multiple comparisons test (E,F).

**
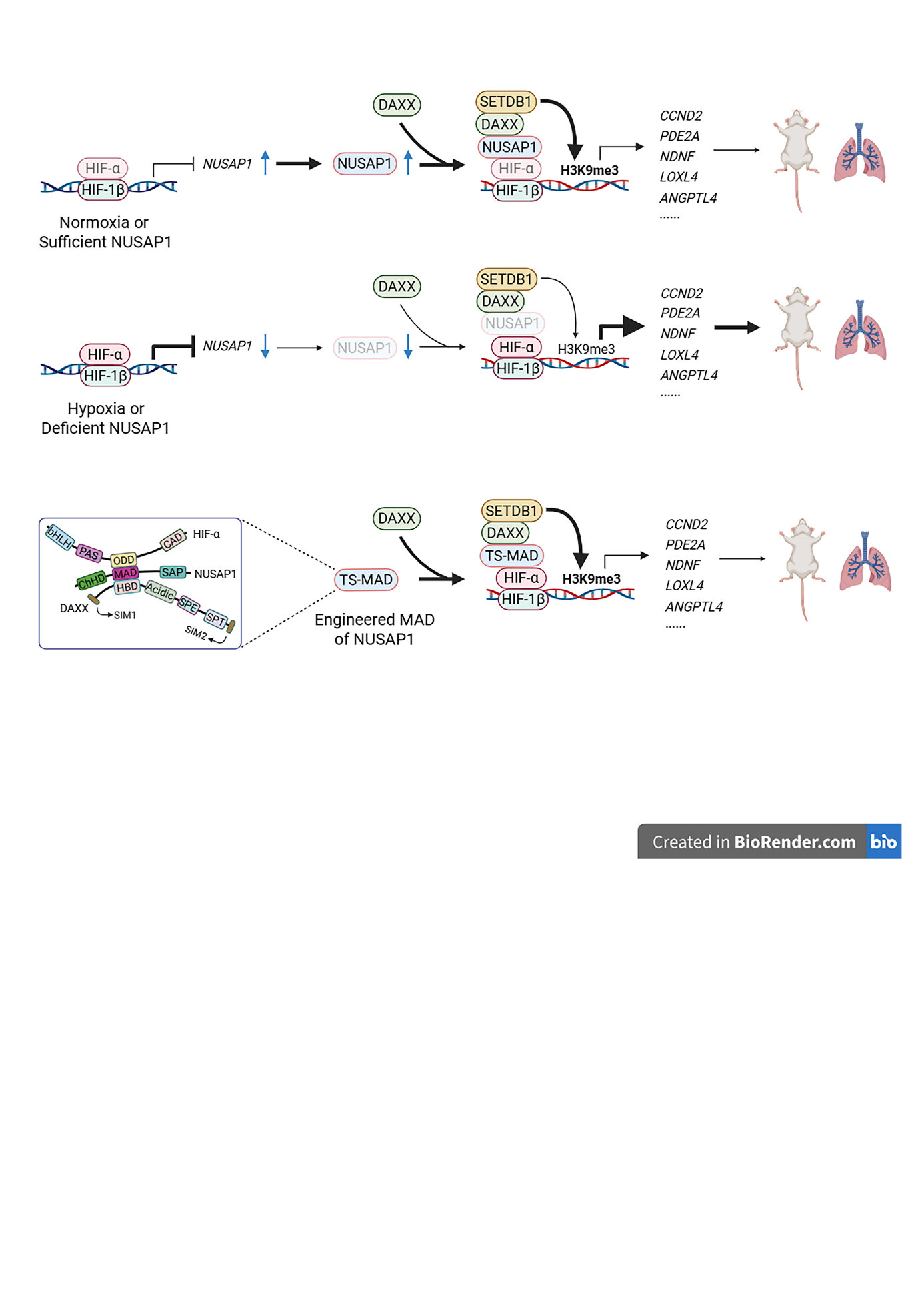
**

**Figure S8. Schematic model of the NUSAP1-HIF double-negative feedback loop in TNBC progression.** Even under normoxic conditions, low levels of HIFα protein persist in TNBC. In this state, relief of HIFα-mediated repression allows NUSAP1 transcriptional accumulation. NUSAP1 then bridges HIF and DAXX via its microtubule-associated domain (MAD) to recruit the methyltransferase SETDB1. The NUSAP1-DAXX-SETDB1 complex represses HIF transcriptional activity by depositing the H3K9me3 repressive mark on hypoxia-response elements (HREs), thereby downregulating HIF target genes and significantly mitigating TNBC tumor growth and metastatic progression. In the hypoxic tumor microenvironment, stabilized HIFα protein actively suppresses NUSAP1 expression. This disrupts the formation of the NUSAP1-DAXX-SETDB1 complex, leading to unrestrained HIF transcriptional activity and driving TNBC progression. The engineered Tumor Suppressor-MAD (TS-MAD) miniprotein specifically enhances the NUSAP1-DAXX-SETDB1 axis-mediated H3K9me3, effectively restoring the suppression of HIF-mediated transcriptional activity even under hypoxic conditions and potently inhibiting TNBC progression.
